# Highly Efficient Recyclable Sol Gel Polymer Catalyzed One Pot Difunctionalization of Alkynes

**DOI:** 10.3390/molecules23081879

**Published:** 2018-07-27

**Authors:** Justin Domena, Carlos Chong, Qiaxian R. Johnson, Bhanu P. S. Chauhan, Yalan Xing

**Affiliations:** 1 Department of Chemistry, William Paterson University of New Jersey, 300 Pompton Rd, Wayne, NJ 07470, USA; domenaj@student.wpunj.edu (J.D.); chongc@student.wpunj.edu (C.C.); 2 Engineered Nanomaterials Laboratory, Department of Chemistry, William Paterson University of New Jersey, 300 Pompton Rd, Wayne, NJ 07470, USA; johnsonq6@wpunj.edu

**Keywords:** green catalysis, sol gel based catalysts, bromination, alkyne, halo-functionalization, one-pot, organosilane

## Abstract

Amino-bridged gel polymer **P1** was discovered to catalyze alkyne halo-functionalization in excellent yields, regioselectivity, functional group compatibility, and recyclability. We have observed that both aromatic and aliphatic alkynes can be converted to α,α-dihalogenated ketones in the presence of polymer **P1** under metal-free conditions at room temperature within a short reaction time.

## 1. Introduction

**Scheme 1:**
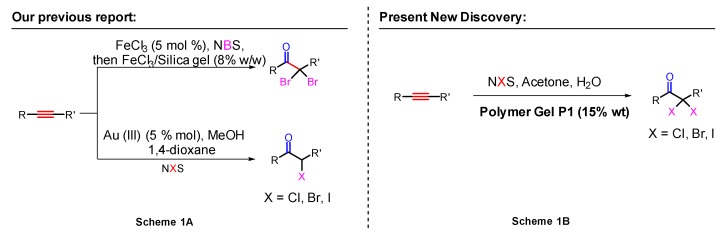
(**1A**) Alkyne difunctionalization and (**1B**) alkyne difunctionalization catalyzed by polymer gel.

Alkyne difunctionalization reactions, the addition of two functional groups across a triple bond, are an efficient method for converting carbon-carbon triple bonds to valuable structures [1,2,3,4,5]. We have been particularly interested in difunctionalization of alkynes for the selective synthesis of mono- and di-halogenated ketones. Very recently, an efficient halo-functionalization of alkynes was developed by our research lab to selectively prepare α,α-dihalogenated ketones and α-halomethyl ketones using iron and gold catalysis respectively (Scheme 1A) [6,7]. Halogenated ketones are versatle synthetic intermediates in organic synthesis and medicinal chemistry [8]. As part of our continued interest for the development of a more efficient and green alkyne difunctionalization methodology, for instance, without the use of metal catalysts, we were intrigued by the possibility of using functionalized silicon based polymers as heterogeneous catalysts.

This study was spurred by the accidental discovery that in the presence of silica gel, partial halo-functionalization of alkynes took place. This observation led us to investigate the catalytic activity of tailor-made silica based materials. In this communication, we present our findings of amino-bridged silsesquioxanes catalyzed halo-functionalization of alkynes for the synthesis of α,α-dihalogenated ketones (Scheme 1B). This transformation was found to furnish an excellent yield of desired α,α-dihalogenated ketones in high selectivity at ambient temperature under 40 min of reaction time. The polymer catalyst was also found to exhibit excellent recyclability. Bridged silsesquioxanes containing a nitrogen bridge have been the materials of interests as catalyst supports due to their complexing abilities with a variety of species [8,9,10,11,12,13]. It is known that metal complexes can coordinate with nitrogen containing silsesquioxanes to furnish heterogeneous recyclable catalysts [14,15,16,17,18]. While looking for an economical and green process for alkyne difunctionalization reactions, we wanted to explore the use of nitrogen containing silica materials as catalysts. This reasoning is based on the fact that the bridged silsesquioxanes containing a nitrogen group can act as an “electrophile directing channel” and may lead to better specificity [19,20,21,22,23,24].

To examine this hypothesis, we utilized polysilsesquioxanes obtained after the hydrolytic polycondensation of bis[3-(trimethoxysilyl)propyl] amine. The silane used in this process contains Si-alkoxy groups at both ends and can undergo hydrolytic polycondensation reactions to provide nitrogen functionalized silica materials. The gelification of bridged silanes is a very well-known process and there have been various studies in which highly cross-linked silica has been produced under various reaction conditions in presence of an acid or base catalyst [25]. The gelification reactions have been carried in the presence or in absence of a co-gelifying agent such as tetraalkoxy silanes.

## 2. Results

**Scheme 2:**
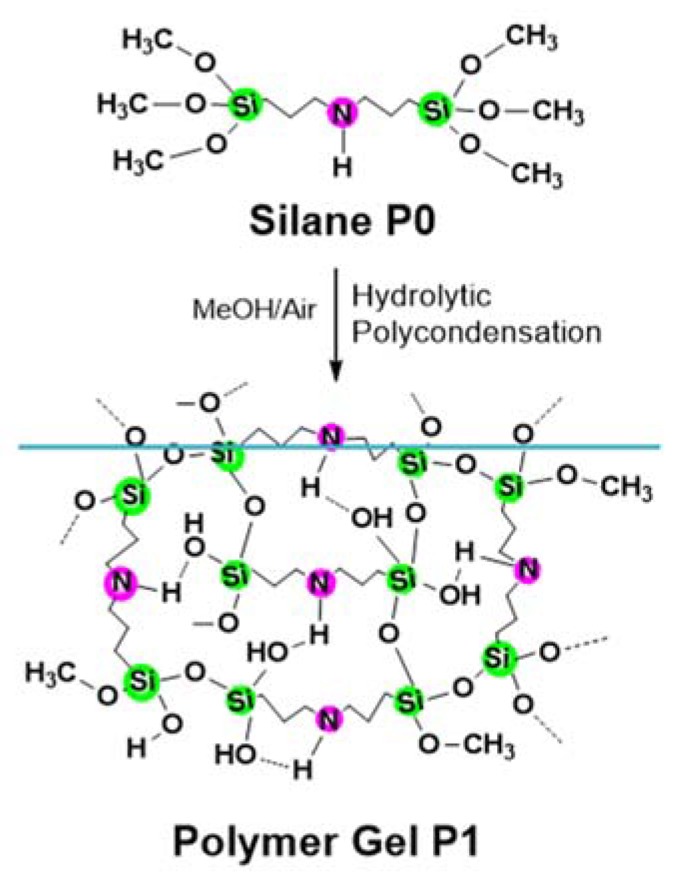
Synthetic strategy to amino-bridged gels.

In terms of catalyst preparation, our goal was to preserve a favorable amount of alkoxy groups on silicon to produce methoxy substituted polycondensates which are known to possess the possibility of “self-healing” via cross linking reactions in water mediated reactions [21,23]. Due to this reason cogelifying agents were avoided. After using known protocols and other variations, we found that, the hydrolytic polycondensation of the precursor bis[3-(trimethoxysilyl)propyl]amine in wet methanol for six hours under ambient reaction conditions and in open air produces colorless silica gel, **P1,** which still has a significant amount of Si-alkoxy groups (Scheme 2). At the initial addition of the solvent to precursor bis[3-(trimethoxysilyl)propyl]amine, there appears to be no visible change. Times for gel formation range from 6 h for 1 mmol and 16 to 22 h for 5 mmol of the reactants.

**Figure 1:**
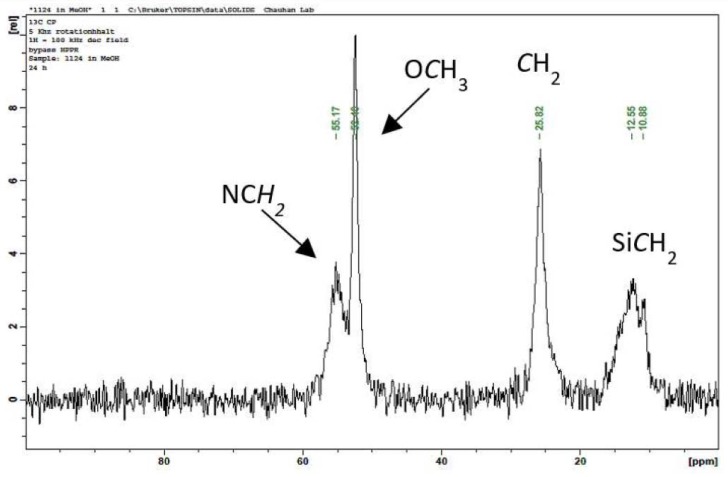
Solid state ^13^C-NMR of the amino-bridged gel **P1**.

Over the course of gelification, NMR spectroscopy shows a small reduction of Si–O–CH_3_ protons indicating a formation of gel through the polymerization and condensation networking of P0 via the formation of Si–OH bonds leading to creation of Si–O–Si networks and methanol. The structural characteristics of the resulting gels were verified by various techniques (see supplementary materials). The solid state NMR studies clearly (Figure 1) showed the presence of residual Si–OCH_3_ groups in resulting silsesquioxanes gel **P1**. The characteristic chemical shifts were similar to liquid phase NMR data of the precusorsor. The residual Si–O–CH_3_ carbon appears at 52.40 ppm as a sharp peak. The carbon to the nitrogen appears slightly further downfield at 55.17 ppm. The signal due to carbon to silicon and nitrogen appears at 25.82 ppm. The carbon to silicon appears as a broad peak at 12.55 and 10.88 ppm due to the network interconnectivity of the Si–O–Si framework. The solid state ^29^Si spectra (see supplementry material) further confirmed the structural assignments. The three peaks −47.26, −56.52, and −64.47 were observed in ^29^Si NMR.The peak centered at −47.26 is assigned to Si(T^0^) atoms confirming the residual Si–O–CH_3_ groups. Thepeak centered at −56.52 ppm was assigned to Si(T^1^) atoms and the peak at −64.4 ppm corresponds to Si(T^2^) atoms. After thorough analysis of the catalyst gel, we started our catalytic investigation by using phenyl acetylene **1a** as the model substrate to optimize reaction conditions. The reaction was conducted with the integration of an organosilane polymer **P1** as a catalyst and *N*-bromosuccinimide (NBS) as an electrophilic bromine source (Table 1). Consistently, the reaction progress was both scrutinized and monitored using TLC and GC-MS.

After a period of intensive investigation, the desired product α,α-dibromoketone **2a** was obtained in both good yield and excellent selectivity over the tri-substitution product **3** when acetone was used as the solvent (entry 4). It was found that 1, 4-dioxane and THF as solvent only produced undesired product **3** and no desired product **2a** was observed (entry 1 and 3). Acetonitrile gave less yield and worse selectivity of the desired product compared to acetone (entry 2). Then the amount of additive H_2_O was increased from 5 equivalents to 10 and 50 equivalents in acetone to improve both yield and selectivity. To our delight, the utility of polymer P1 in the presence of 50 equivalennts of H_2_O in acetone at room temperature was found to be the optimal conditions (entry 6) which generated the desired product in 90% yields and gave high regioselectivity (90:8).

We noticed that these results provided a clear indication of two major driving forces within our one-pot reaction: both the necessity of water and the introduction of the sol-gel catalyst. To confirm the importance of these two factors, we then decided to run a reaction without both water and gel (entry 9). Not surprisingly, the reaction did not produce any products. Next, two control reactions were run with regular silica gel (entry 7) and no polymer catalyst applied (entry 8). Both reactions resulted with a decreased yield and regioselectivity of the major product. These results confirmed the importance of our polymer catalyst **P1** in terms of the yield and regioselectivity of this transformation.

**Figure 2:**
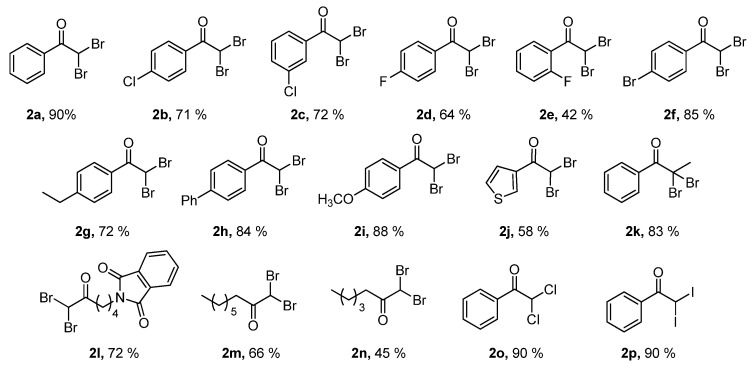
Substrate scope with isolated yields.

With optimal conditions (entry 6, Table 1) in hand, the next phase initiated was the study of substrate scope and limitation shown in Figure 2. In general, this reaction converts l alkynes to α,α-dihalogenatedketones in good isolation yields. NBS (**2a**–**2n**), NCS (*N*-chlorosuccinimide) (**2o**), and NIC (*N*-iodosuccinimide) (**2p**) were utilized as the halogen sources and all showed excellent regioselectivity as well. Also, this reaction exhibits good functional group compatibility. Aromatic compounds with a variation in both electron-donating and electron-withdrawing groups on the benzene in different positions were explored. Substrates with halogens including: chlorine, bromine, and fluorine on the different positions of the phenyl ring gave good to excellent yields (**2b-2f**). It is also worth noting that strong electron-withdrawing fluorine groups showed decreased reaction yields (**2d** and **2e**). In addition, alkyl substations such as 4-ethynyl-1,1′-biphenyl, 1-ethyl-4-ethynylbenzene, and 1-ethynyl-4-methoxybenzene produced a 72% (**2h**) yield, 84% (**2g**) yield, and 88% (**2i**), respectively. Heterocyclic rings were also tolerated in this reaction condition and generated the desired products in good yields (**2j** and **2l**). In addition, not only terminal alkynes, but also an internal alkyne (**2k**) underwent this reaction smoothly and gave good yield and regioselectivity of the desired product. In which our reaction then proceeded to explore two aliphatic compounds; 1-nonyne and 1-heptyne. These two compounds were then observed to give acceptable yields of 66% (**2m**) and 45% (**2n**). Moreover, NCS and NIS also participated this transformation as halogen sources to give α,α-dichloroketone (**2o**) and α,α-diiodoketone (**2p**) in excellent yields.

**Figure 3:**
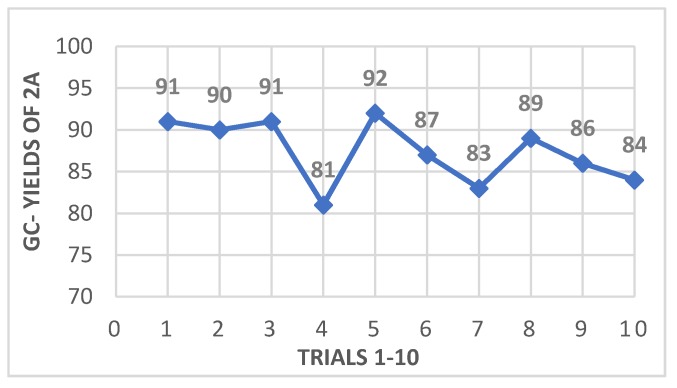
Recyclability studies.

In order to further demonstrate the applicability of this gel catalyzed transformation, we tested the recyclability of the catalyst under optimized reaction conditions. The transformation outlined in entry 6 of Table 1 was used as standard protocol. To our delight, **P1** was found to maintain similar GC-conversion even after ten cycles (trial 10). A graphical representation with GC yields is shown in Figure 3. This study clearly bodes well for the real world applications of our present catalytic system.

The detailed mechanistic studies are ongoing in our laboratories and will be presented in due course. At this stage, based on the present results and the results of our previous study, we believe that the use of the sol-gel polymer as a catalyst significantly increses the electrophilicity of NBS. The increased electrophilicity is achieved via interaction of nitrogen of gel catalyst P1 (Scheme 2) with the NBS bromine. This additional N–Br interaction increases the electrophilicity of Br and helps the efficient delivery of Br+ in a highly regioselective manner.

## 3. Conclusions

In conclusion, we have revealed a very simple methodology to produce cross linked polymer **P1** using bis[3-(trimethoxysilyl)propyl] amine as precursor which was found to catalyze the one-pot reaction of alkyne halo-functionalization to synthesize α,α-dihalogenated ketones. To our satisfaction, our sol-gel catalyst **P1** was then found to induce good reaction yields, to proceed under ambient temperatures, and to exhibit excellent catalyst recyclability. The implications of this discovery are promising in expanding the field of green chemistry centered synthesis via the utilization of a green catalyst; as well as expanding the field of polymer chemistry pertaining to catalysis within an organic synthesis reaction. Future studies of this phenomenon are currently being conducted within our laboratory to further expand the scope of sol-gel catalysts utilized as well as the implementation of the sol-gel catalysts within more complex organic synthetic methods.

## Figures and Tables

**Table 1:**
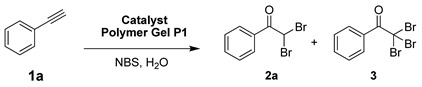
Optimization of reaction conditions for the synthesis of α,α-dibromoketones.

Entry ^[a]^	Catalyst	H_2_O Equiv.	Solvent (1.25 mL)	Yield ^[b]^	
				2a	3
1	Gel P1	5	1,4 Dioxane	0	16
2	Gel P1	5	Acetonitrile	6	23
3	Gel P1	5	THF	0	24
4	Gel P1	5	Acetone	45	5
5	Gel P1	10	Acetone	53	6
6	Gel P1	50	Acetone	90	8
7	Silica gel ^[c]^	50	Acetone	68	10
8	No	50	Acetone	78	10
9	No	0	Acetone	0	0

^[a]^ Reaction condition: phenyl acetylene (0.5 mmol), NBS (2.00 mmol), solvent (3 mL), and catalyst (15 wt. %) at room temperature for 40 min. ^[b]^ GC yield. ^[c]^ Silica gel 60 (230–400 mesh).

## Supplementary Materials

The following are available online, General procedure; preparation and characterization of polymer catalyst **P1**; characterization of ^1^H and ^13^C NMR spectra of α,α-dihalogenated ketones.
