# Supplementary material for: Highly Efficient Recyclable Sol Gel Polymer Catalyzed One Pot Difunctionalization of Alkynes
Source: Molecules. 2018 Jul 27;23(8):1879. doi: 10.3390/molecules23081879 (PMC6222667; doi:10.3390/molecules23081879)
Supplement: Supplementary file 1 [file molecules-23-01879-s001.pdf]

Experimental Section

Section A: General Information

General methods and materials: ^{1}H and ^{13}C spectra were recorded on a Bruker 400 MHz spectrometer. Chemical shifts were reported relative to internal tetramethylsilane (δ 0.00 ppm) or $CDCl_3$ (δ 7.26 ppm) for ^{1}H and $CDCl_3$ (δ 77.0 ppm) for ^{13}C. Infrared (IR) spectra were obtained on a Thermo Nicolet iS5 FT-IR spectrometer. GC-MS data were obtained on a Thermo Scientific CG Mass Machine and were reported in units of m/z. Flash column chromatography was performed on silica gel 60 (230-400 mesh). Analytical thin-layer chromatography was performed with precoated glass-backed plates (K6F 60Å, F_{254}) and visualized by quenching of fluorescence and by charring after treatment with *p*-anisaldehyde or potassium permanganate stain. Commercial reagents were used without purification.

Section B: Experimental Procedures

1. **General procedure for synthesis of α,α-dibromoketone using Sol Gel Polymer P1**

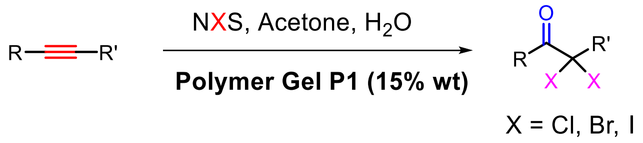

To a solution of alkyne (0.5 mmol), NXS (2.00 mmol), acetone (3 mL), H_2O (2.5 mmol) and Sol Gel polymer (15% wt.). The reaction solution was stirring 40 min at room temperature. When TLC showed full consumption of starting material, reaction was placed in a centrifuged tube. Supernatant was separated from the Sol Gel polymer and filtered. Filtrate was collected and solvent was removed using the rotary evaporator. The mixture was purified by flash chromatography on Silica Gel 60 to give pure product.

Section C. Preparation and Characterization of Polymer catalyst P1

Synthetic Procedure:

In a tarred 20 mL scintillation vial, 1 mmol (0.341 g) of 3,3′-bis(trimethoxysilyl)dipropylamine (**P0**) was weighed and a 25 x 6 mm magnetic stirrer bar added. The flask was then placed on a stirrer/hot plate to stir at moderate intensity with the top loosely covering. While stirring, 2 mL of wet methanol (used as received) was slowly added using a 1 mL glass syringe. The resulting mixture was left open with only stirring under a vent hood until a gel formed which, depending on volume and ambient conditions, took up to 6 hours. Within five minutes after the precursors were added, initial spectroscopic readings were taken by using a micropipette to remove 350 µL of sample material for NMR and 3 µL for FT-IR. Readings were taken every 2 hours. The gel sample was left to further dry for an additional 24 hours. At this point gel was washed with another 2 mL of methanol by stirring with a 12 x 4.5 mm stirrer bar at a high rate. The suspension was centrifuged and the solution was decanted to provide off white powder gel. This gel sample **P1** was analyzed using FT-IR, NMR, TEM and SEM. The Product P1 was then used for catalysis.

Analytical data for P0: FT-IR ν(cm^{-1}): 2937 (m), 2838 (m), 1188 (m), 1080 (vs), 776 (s). **NMR**: ^{13}C Cross Polarization NMR (400 MHz, $CDCl_3$,δ) 51.97 (C_1), 40.57 (CH_3), 22.74 (C_2), 6.15 (C_3). ^{29}Si NonCoupled (400 MHz, $CDCl_3$,δ) -42.17 $Si(OCH_3)_3$.

Analytical data for P1: FT-IR ν(cm^{-1}): 2923 (s), 2872 (s), 1472 (w), 1409 (w), 1004 (vs), 913 (s), 686 (m); ^{13}C Cross Polarization SSNMR (400 MHz, 100 kHz dec field, 5 kHz rotation, δ) 55.17 (C_1), 52.40 (CH_3), 25.82 (C_2), 12.55 and 10.88 (C_3). ^{29}Si NonCoupled SSNMR (400 MHz, 100 kHz dec field, 5 kHz rotation, δ) -47.26 mixture of SiOH and $Si(OCH_3)$ -56.52 difunctional SiO, -64.47 trifunctional SiO.

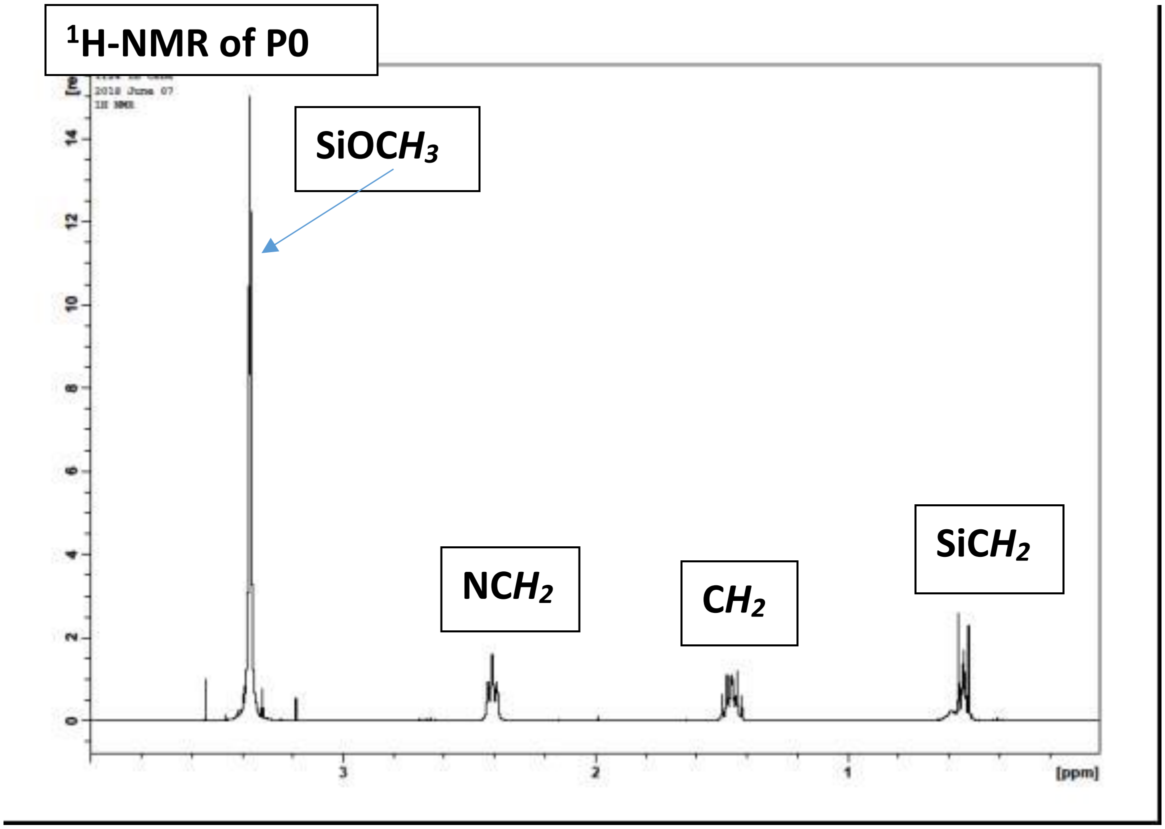
1H-NMR of P0
SiOCH3
NCH2
CH2
SiCH2
[ppm]

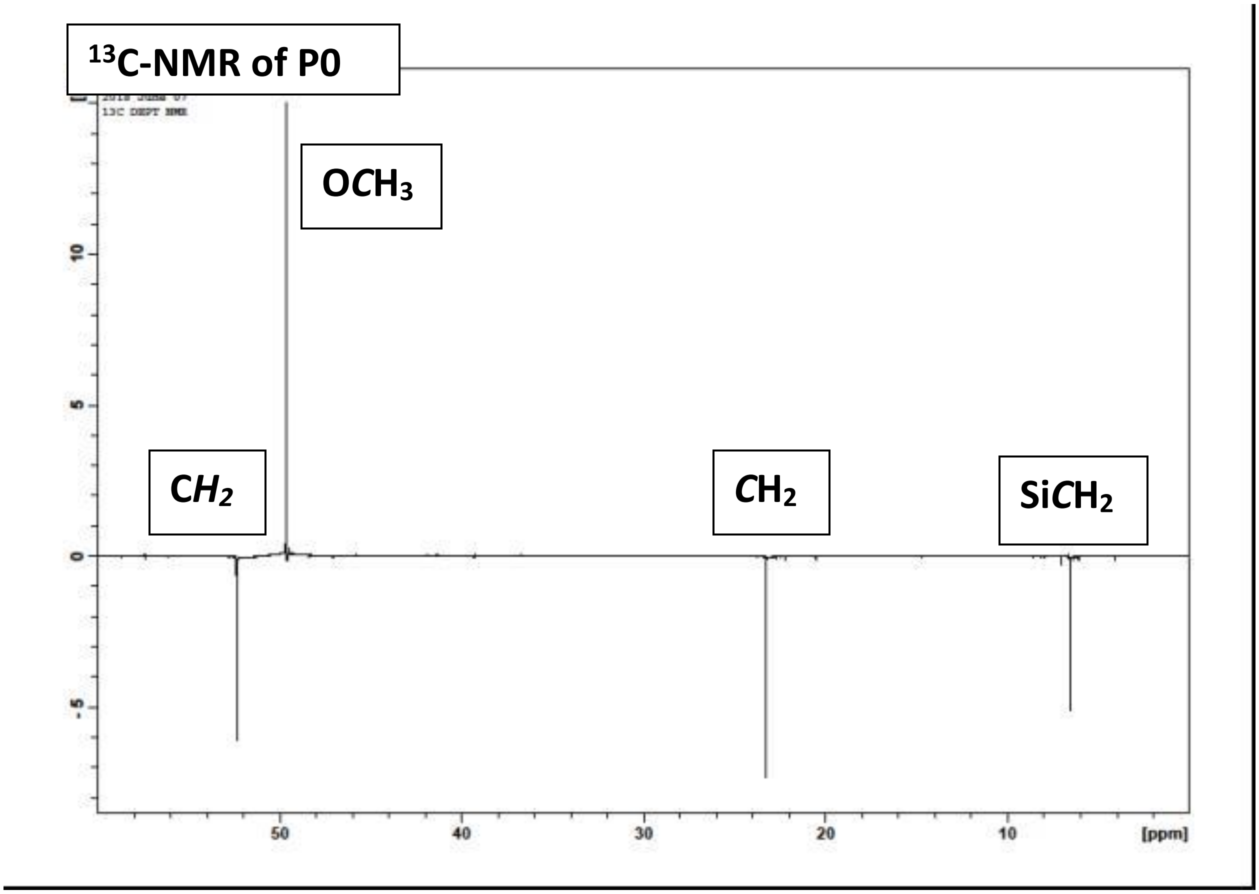
13C-NMR of P0
13C DEPT NMR
OCH3
CH2
CH2
SiCH2
[ppm]

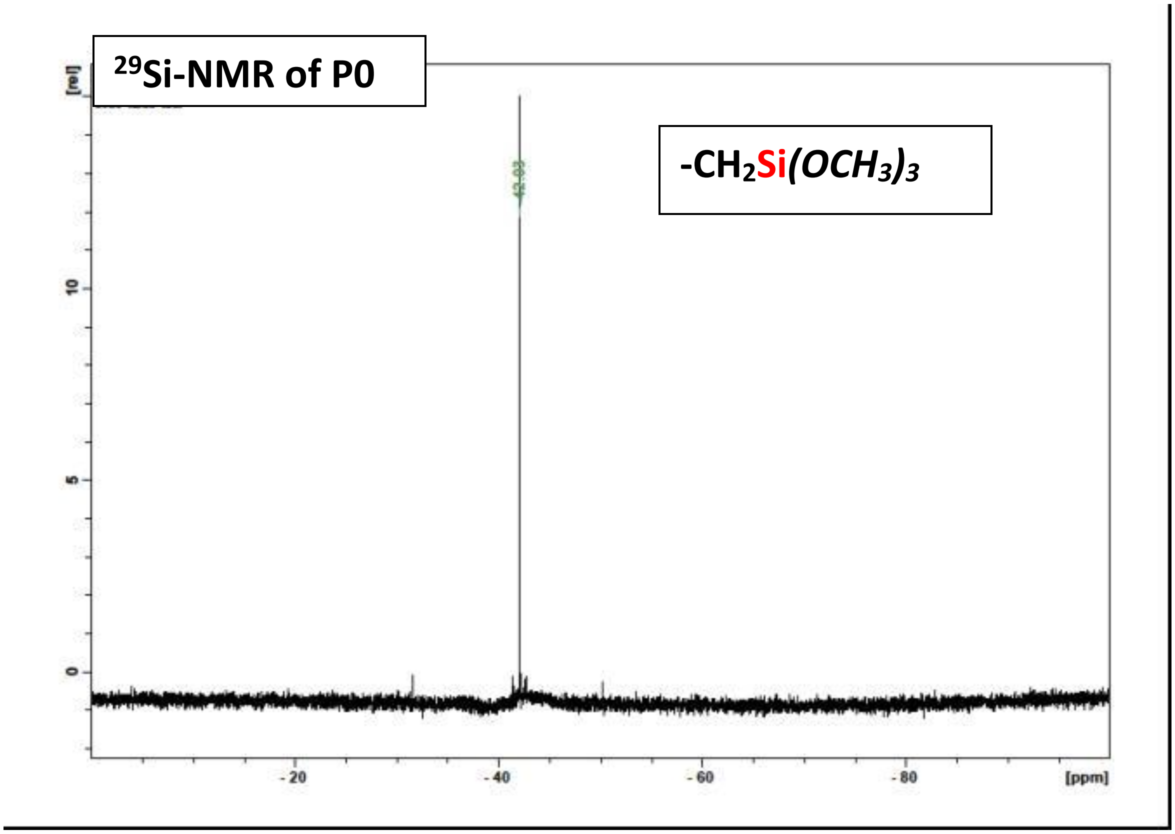

^{29}Si-NMR of P0
-CH_2Si(OCH_3)$_3$
[rel]
0
5
10
- 20
- 40
- 60
- 80
[ppm]

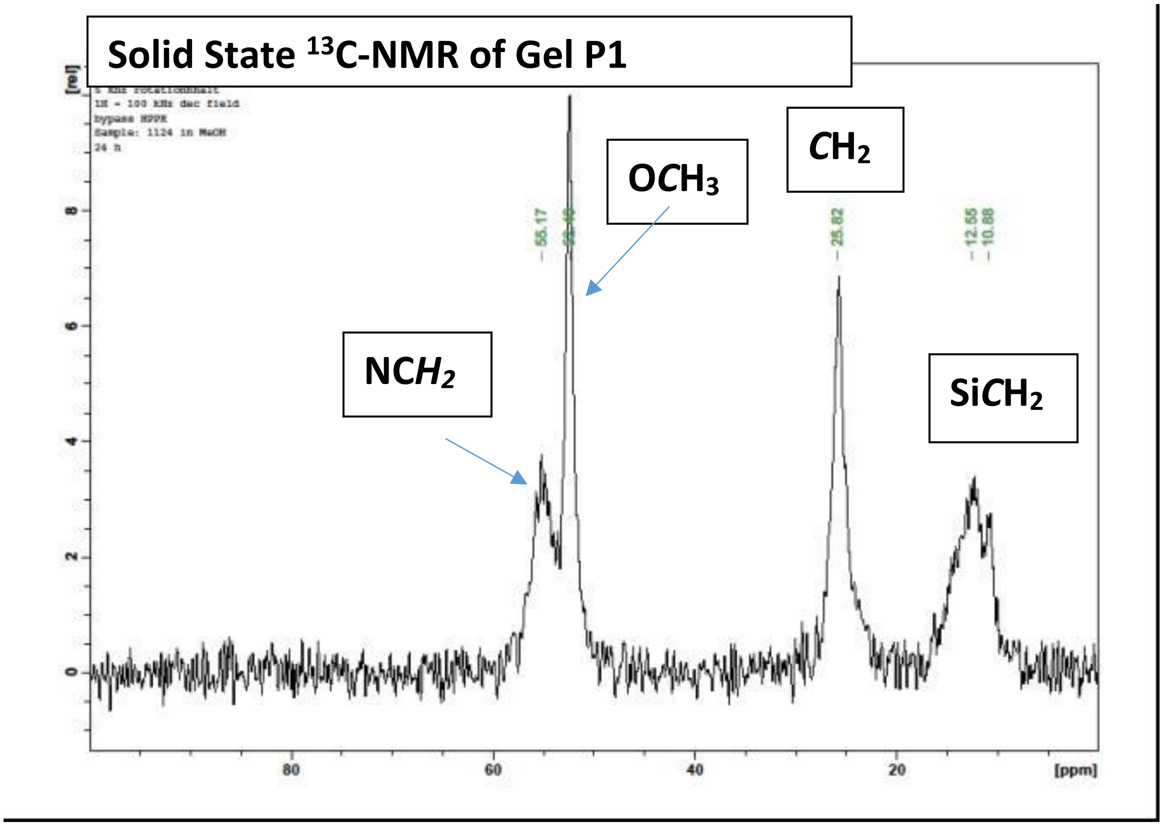

Solid State ^{13}C-NMR of Gel P1
[rel]
OCH_3
CH_2
NCH_2
$SiCH_2$
55.17
25.82
12.55
10.88
0
2
4
6
8
80
60
40
20
[ppm]

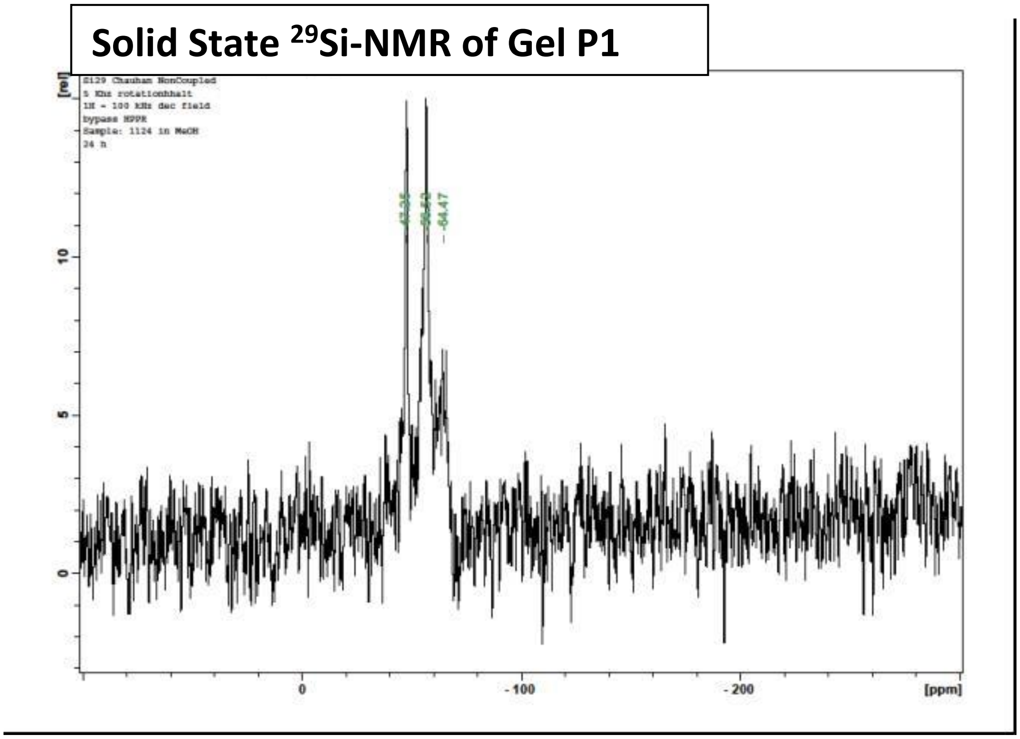

Solid State ^{29}Si-NMR of Gel P1

Section D: Characterization and ^{1}H and ^{13}C NMR spectra of α,α-dihalogenated ketones

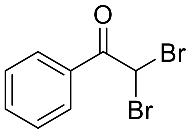

2a

^{1}H NMR (400 MHz, $CDCl_3$) δ 8.10 -8.07 (m, 2 H), 7.66 -7.62 (m, 1 H), 7.53 -7.49 (m, 2 H), 6.72 (s, 1 H). ^{13}C NMR (100 MHz, $CDCl_3$) δ 185.9, 134.4, 130.8, 129.6 (2 C), 128.9 (2 C), 39.6; GC-MS m/z (relative intensity) 51 (13), 63 (5), 77 (33), 89 (6), 90 (9), 105 (100), 106 (7), 171 (3). Clear oil.

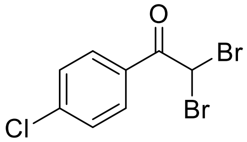

2b

^{1}H NMR (400 MHz, $CDCl_3$) δ 8.05 (d, *J* = 8 Hz, 2 H), δ 7.48 (d, *J* = 8 Hz, 2 H), δ 6.62 (s, 1 H); ^{13}C NMR (100 MHz, $CDCl_3$) δ 184.9, 141.1, 131.2 (2C), 129.3 (2C), 129.1, 39.2; GC-MS m/z (relative intensity) 51 (14), 77 (47), 105 (100), 106 (8), 125 (4). White solid, mp 93-94 °C.

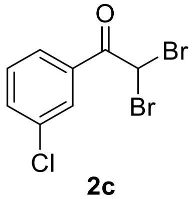

2c

^{1}H NMR (400 MHz, $CDCl_3$) δ 8.07 -8.06 (m, 1 H), δ 7.99 -7.97 (m, 1 H), δ 7.62 -7.60 (m, 1 H), δ 7.46 (t, *J* = 8 Hz, 1 H), δ 6.62 (s, 1 H); ^{13}C NMR (100 MHz, $CDCl_3$) δ 184.8, 135.3, 134.4, 132.4, 130.2 129.8, 127.8, 39.1; GC-MS m/z (relative intensity) 50 (4), 51 (19), 73 (5), 77 (55), 84 (7), 105 (100), 106 (9), 159 (2). Light yellow oil.

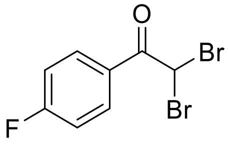

2d

^{1}H NMR (400 MHz, $CDCl_3$) δ 8.17 -8.13 (m, 2 H), δ 7.20 -7.16 (m, 2 H), δ 6.63 (s, 1 H). ^{13}C NMR (100 MHz, $CDCl_3$) δ 184.5, 167.6, 165.0, 132.7, 132.6, 116.3, 116.1, 39.3; GC-MS m/z (relative intensity) 57 (4), 74 (4), 75 (17), 94 (5), 95 (43), 107 (12), 108 (15), 123 (100), 124 (10), 187 (7), 189 (7). Light yellow oil.

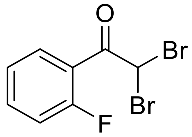

2e

^{1}H NMR (400 MHz, $CDCl_3$) δ 8.01- 7.96 (m, 1 H), δ 7.65- 7.59 (m, 1 H), δ 7.33- 7.29 (m, 1 H), δ 7.21- 7.16 (m, 1 H), δ 6.85 (d, *J* = 8 Hz, 1 H). ^{13}C NMR (100 MHz, $CDCl_3$) δ 184.2, 162.2, 159.7, 136.1, 132.4, 125.2, 116.9, 43.0; GC-MS m/z (relative intensity) 87 (3), 73 (3), 75 (12), 94 (4), 95 (29), 107 (8), 108 (11), 123 (100), 124 (8), 186 (3), 189 (3). Light yellow oil.

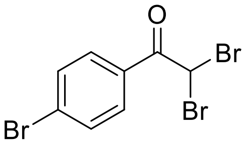

2f

^{1}H NMR (400 MHz, $CDCl_3$) δ 7.96 (d, J = 8 Hz, 2 H), δ 7.65 (d, J = 8 Hz, 2 H), δ 6.60 (s, 1 H). ^{13}C NMR (100 MHz, $CDCl_3$) δ 185.1, 132.3 (2 C), 131.2 (2 C), 129.9, 129.5, 39.2; GC-MS m/z (relative intensity) 50 (39), 51 (12), 62 (9), 63 (16), 74 (30), 75 (48), 76 (42), 89 (22), 90 (28), 93 (9), 95 (12), 123 (11), 155 (27), 157 (26), 169 (8), 171 (8), 183 (93), 184 (9), 185 (100). White solid, mp 89-90 °C

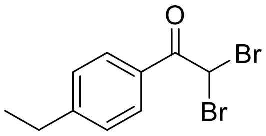

2g

^{1}H NMR (400 MHz, $CDCl_3$) δ 8.01 (d, J = 8 Hz, 2 H), δ 7.33 (d, J = 8 Hz, 2 H), δ 6.71 (s, 1 H), δ 2.73 (q, J = 8 Hz, 2 H), δ 1.27 (t, J = 8 Hz, 3 H). ^{13}C NMR (100 MHz, $CDCl_3$) δ 185.6, 151.8, 132.2, 129.9, 128.4, 39.9, 29.0, 15.0; GC-MS m/z (relative intensity) 51 (5), 63 (4), 77 (12), 79 (9), 89 (5), 103 (9), 105 (11), 117 (7), 118 (6), 133 (100), 134 (11). Light yellow oil.

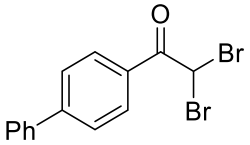

2h

^{1}H NMR (400 MHz, $CDCl_3$) δ 8.19 -8.16 (m, 2 H), δ 7.75 -7.72 (m, 2 H), δ 7.66 -7.63 (m, 2 H), δ 7.51 -7.41(m, 3 H), δ 6.73 (s, 1 H). ^{13}C NMR (100 MHz, $CDCl_3$) δ 185.6, 147.2, 139.4, 130.4, 129.1 (4 C), 127.3 (5 C), 39.7; GC-MS m/z (relative intensity) 51 (5), 63 (7), 74 (5), 75 (6), 76 (14), 88 (7), 89 (8), 150 (6), 151 (8), 152 (11), 176 (24), 177 (14), 178 (100), 179 (15). Light yellow oil.

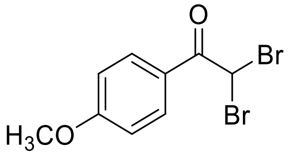

2i

^{1}H NMR (400 MHz, $CDCl_3$) δ 8.07 (d, J = 8 Hz, 2 H), δ 6.97 (d, J = 8 Hz, 2 H), δ 6.67 (s, 1 H), δ 3.90 (s, 3 H). ^{13}C NMR (100 MHz, $CDCl_3$) δ 184.6, 164.5, 132.2 (2 C), 123.4, 114.2 (2 C), 55.7, 39.9; GC-MS m/z (relative intensity) 51 (6), 63 (7), 64 (6), 77 (17), 91 (6), 92 (11), 107 (7), 135 (100), 136 (10), 199 (4), 201 (5). Light yellow oil.

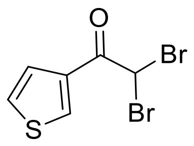

2j

^{1}H NMR (400 MHz, $CDCl_3$) δ 8.37 (s, 1 H), δ 7.67 (d, *J* = 4 Hz, 1 H), δ 7.39 (d, *J* = 8 Hz, 1 H), δ 6.44 (s, 1 H). ^{13}C NMR (100 MHz, $CDCl_3$) δ 180.5, 135.3, 134.53, 128.0, 126.7, 40.2; GC-MS m/z (relative intensity) 69 (4), 81 (4), 82 (12), 96 (9), 111 (100), 112 (7), 113 (5). Light yellow oil.

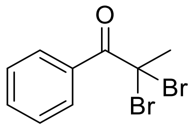

2k

^{1}H NMR (400 MHz, $CDCl_3$) δ 8.41 -8.40 (m, 2 H), δ 7.58 -7.56 (m, 1 H), δ 7.49 -7.44 (m, 2 H), δ 2.76 (s, 3 H). ^{13}C NMR (100 MHz, $CDCl_3$) δ 188.3, 133.5 (2 C), 131.3 (2 C), 128.0 (2 C), 57.9, 37.7; GC-MS m/z (relative intensity) 51 (16), 77 (39), 78 (39), 78 (4), 103 (6), 104 (6), 105 (100), 106 (8). Light yellow oil.

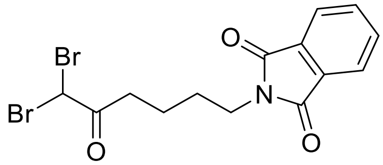

2l

^{1}H NMR (400 MHz, $CDCl_3$) δ 7.84 -7.81 (m, 2 H), δ 7.71 -7.69 (m, 2 H), δ 5.78 (s, 1 H), δ 3.71 (t, *J* = 8 Hz, 2 H), δ 2.99 (t, *J* = 8 Hz, 2 H), δ 1.74 -1.70 (m, 4 H). ^{13}C NMR (100 MHz, $CDCl_3$) δ 196.3, 168.3, 133.9 (2 C), 132.0 (2 C), 123.2 (2 C), 42.7, 37.3, 34.0, 27.6, 21.4; GC-MS m/z (relative intensity) 75 (8), 76 (25), 77 (30), 79 (9), 80 (39), 102 (7), 104 (23), 105 (16), 130 (24), 133 (18), 148 (36), 149 (11), 160 (100), 161 (20), 186 (17), 188 (10), 198 (6), 199 (15), 226 (7). Light yellow oil.

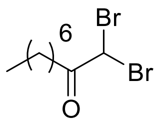

2m

^{1}H NMR (400 MHz, $CDCl_3$) δ 5.78 (s, 1 H), δ 2.92 (t, J = 8 Hz, 2 H), δ 1.67 (t, J = 8 Hz, 2 H), δ 1.32 -1.29 (m, 8 H), δ 0.88 (t, J = 8 Hz, 3 H). ^{13}C NMR (100 MHz, $CDCl_3$) δ 197.0, 43.0, 34.9, 31.6, 28.9 (2 C), 24.4, 22.6, 14.1; GC-MS m/z (relative intensity) 55 (24), 56 (4), 57 (100), 58 (5), 67 (5), 69 (4), 109 (6), 120 (4), 122 (4), 127 (72), 128 (7), 173 (4). Clear oil.

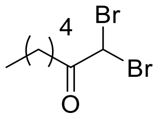

2n

^{1}H NMR (400 MHz, $CDCl_3$) δ 5.78 (s, 1 H), δ 2.92 (t, J = 8 Hz, 2 H), δ 1.70-1.66 (m, 2 H), δ 1.35-1.32 (m, 5 H), δ 0.93-0.89 (m, 3 H). ^{13}C NMR (100 MHz, $CDCl_3$) δ 197.0, 43.0, 34.9, 31.1, 24.0, 22.3, 13.9; GC-MS m/z (relative intensity) 43 (4), 55 (20), 71 (52), 99 (100), 100 (7), 120 (5), 122 (5), 173 (4). Clear oil.

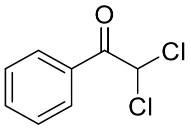

2o

^{1}H NMR (400 MHz, $CDCl_3$) δ 8.10 -8.08 (m, 2 H), δ 7.68 -7.64 (m, 1 H), δ 7.55 -7.51 (m, 2 H), δ 6.69 (s, 1 H). ^{13}C NMR (100 MHz, $CDCl_3$) δ 185.9, 134.5, 131.3, 129.7 (2 C), 128.9 (2 C), 67.8; GC-MS m/z (relative intensity) 52 (4), 53 (4), 54 (50), 55 (100), 69 (33), 79 (6), 97 (11), 98 (98), 99 (6), 104 (7), 106 (4), 132 (41). Clear oil.

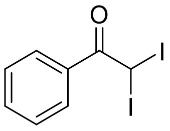

2p

^{1}H NMR (400 MHz, $CDCl_3$) δ 8.11 -8.08 (m, 2 H), δ 7.68 -7.64 (m, 1 H), δ 7.55 -7.50 (m, 2 H), δ 6.69 (s, 1 H). ^{13}C NMR (100 MHz, $CDCl_3$) δ 188.3, 134.1, 139.5 (2 C), 129 (2 C), 128.9, 1.6; GC-MS m/z (relative intensity) 50 (4), 51 (13), 61 (4), 62 (5), 73 (6), 74 (21), 75 (35), 98 (5), 99 (4), 101 (41), 102 (4), 103 (4), 114 (10), 126 (4), 228 (100), 229 (10). Clear oil.

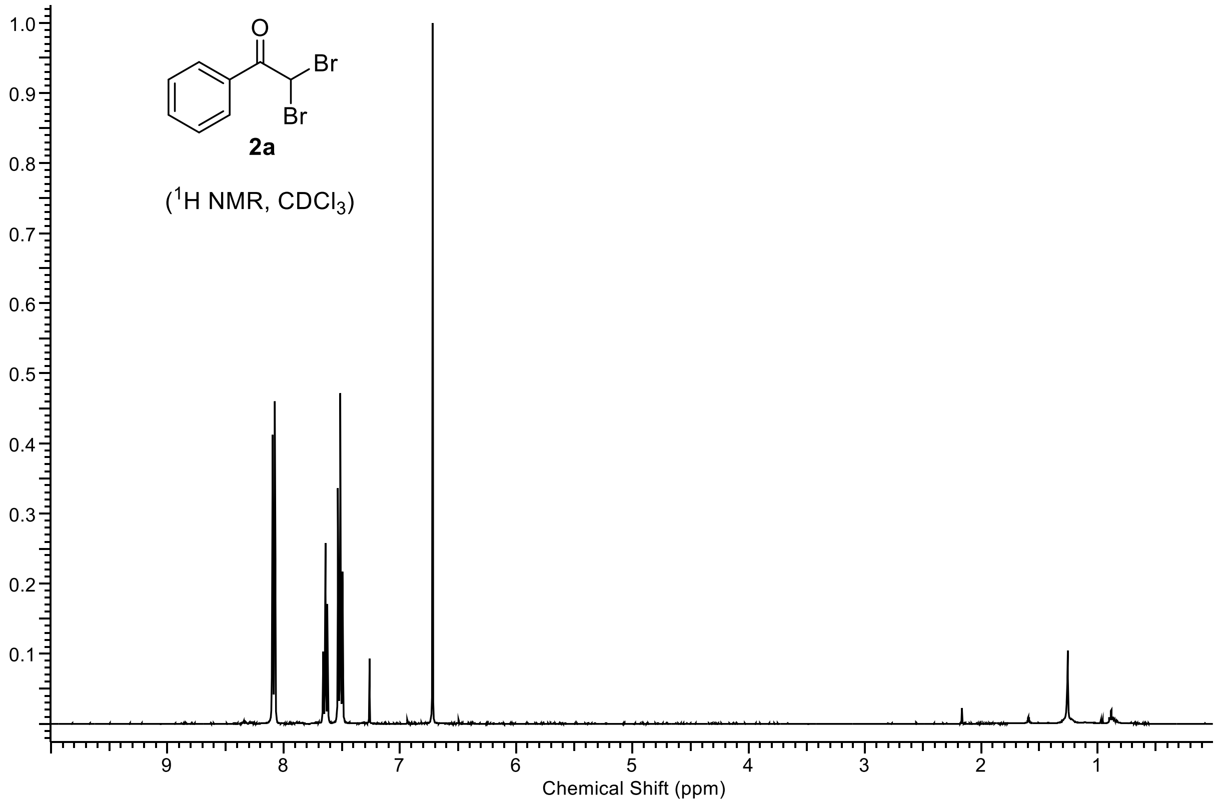
O
Br
Br
2a
(1H NMR, CDCl3)
Chemical Shift (ppm)

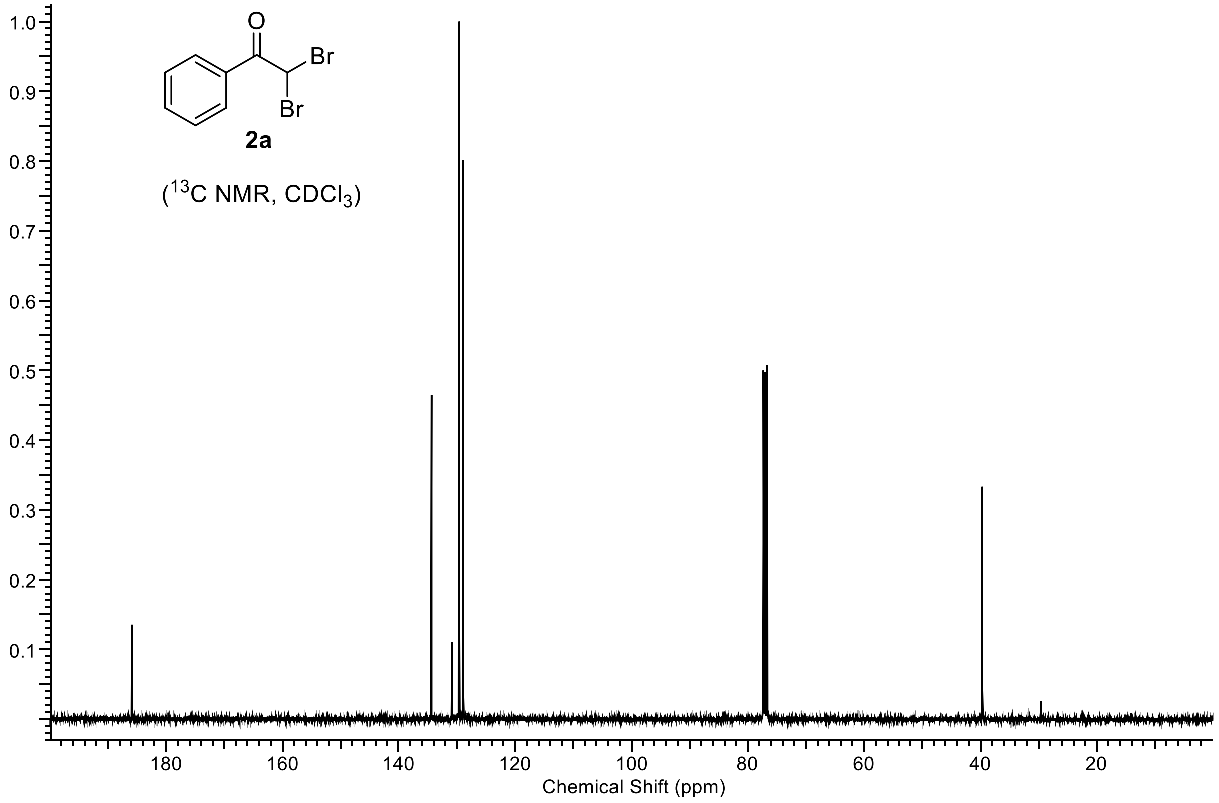
O
Br
Br
2a
(13C NMR, CDCl3)
Chemical Shift (ppm)

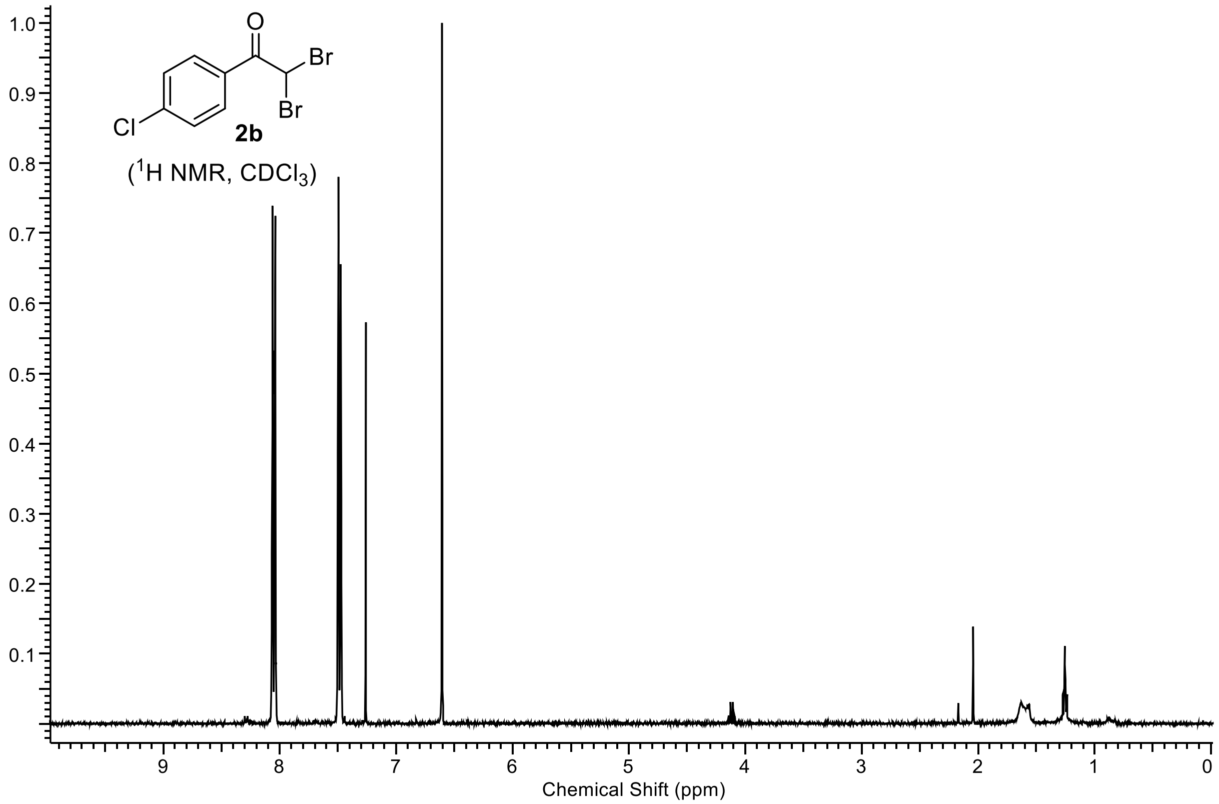
O
Br
Br
Cl
2b
(^{1}H NMR, CDCl$_3$)
Chemical Shift (ppm)

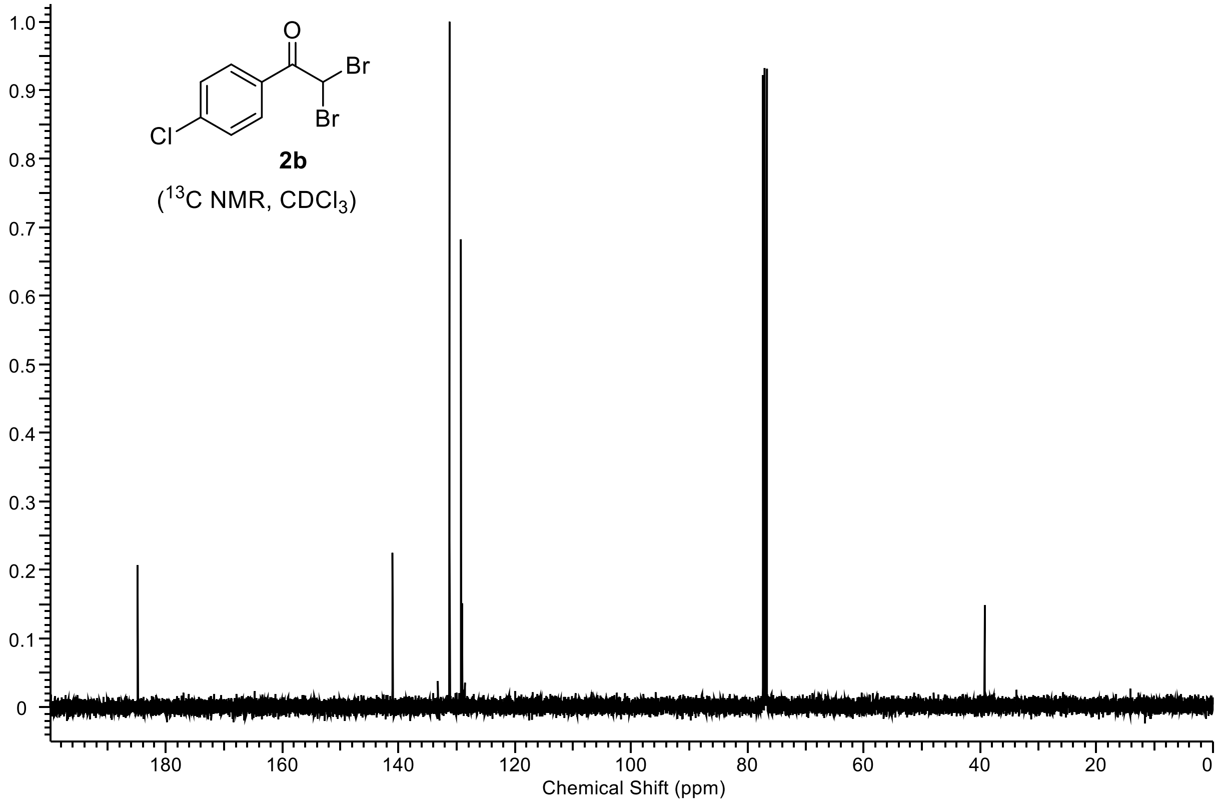
O
Br
Br
Cl
2b
(^{13}C NMR, CDCl$_3$)
Chemical Shift (ppm)

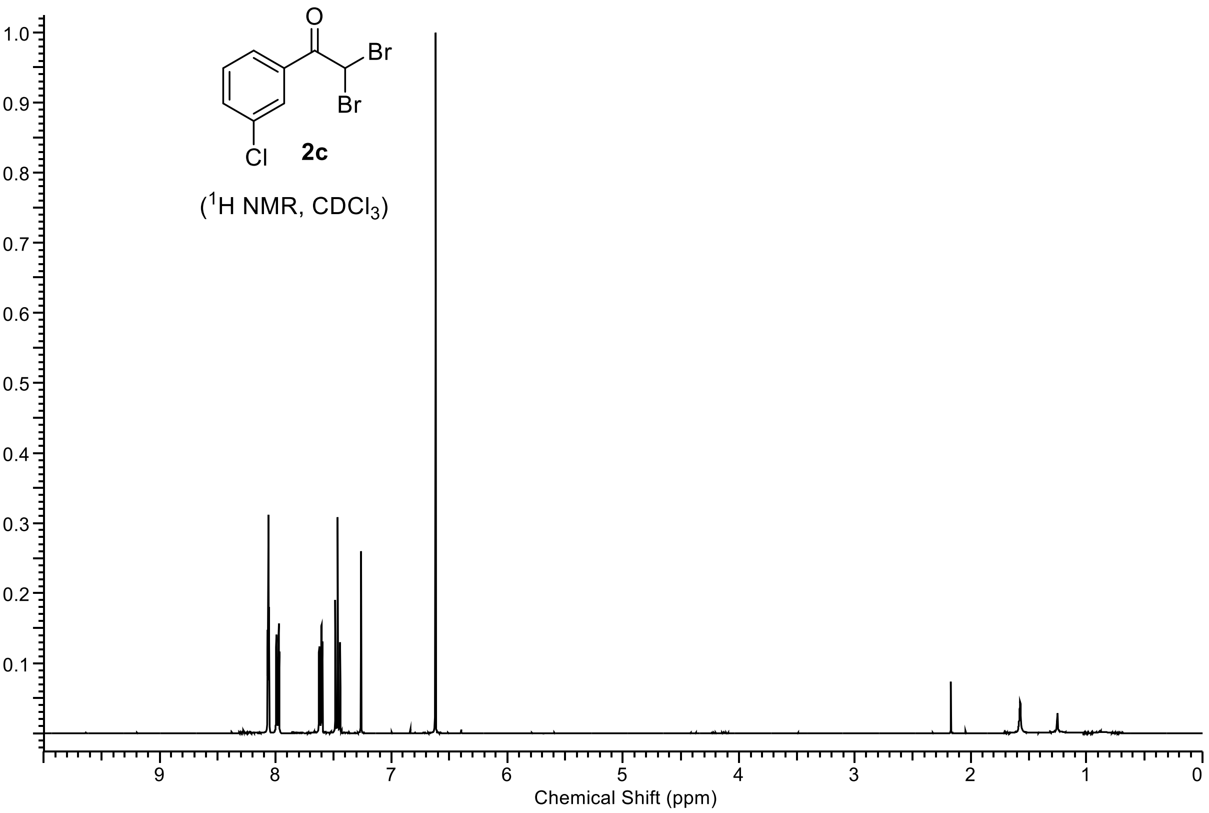

O
Br
Br
Cl
2c
(^{1}H NMR, CDCl$_3$)
Chemical Shift (ppm)

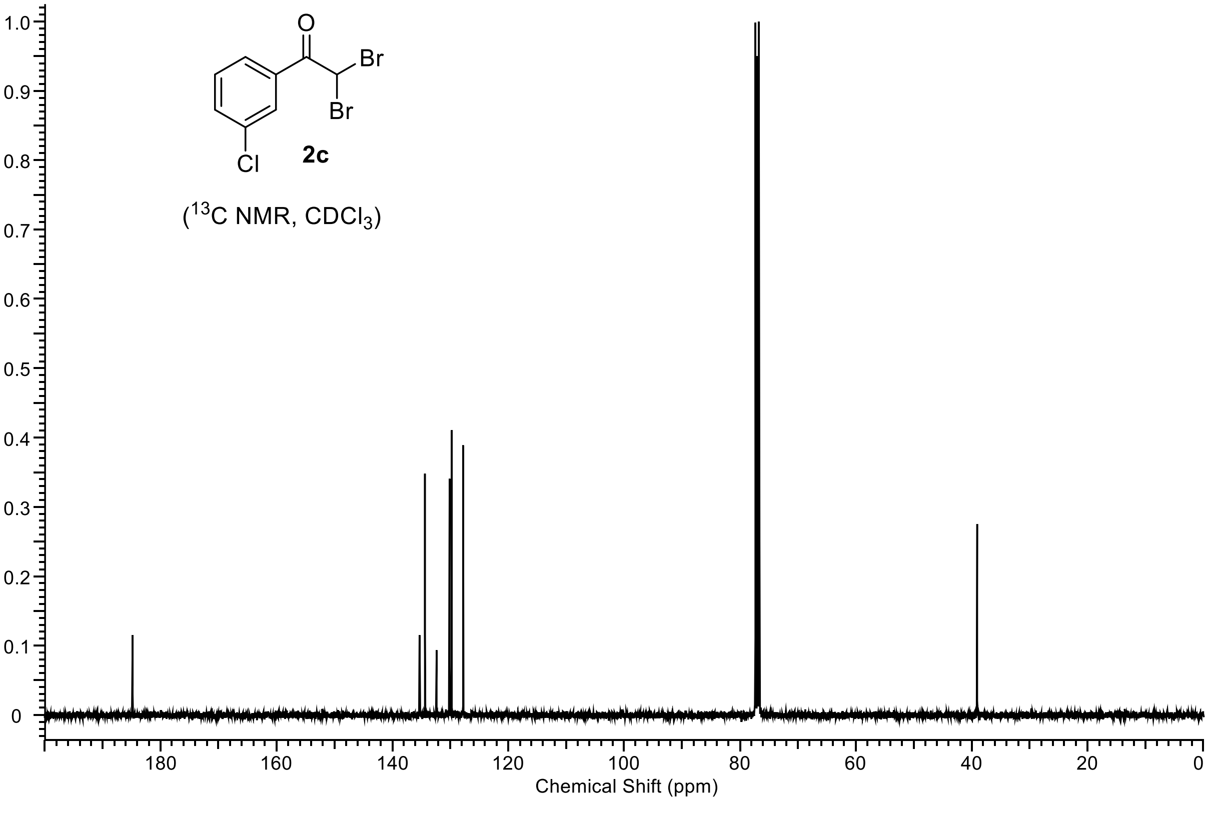

O
Br
Br
Cl
2c
(^{13}C NMR, CDCl$_3$)
Chemical Shift (ppm)

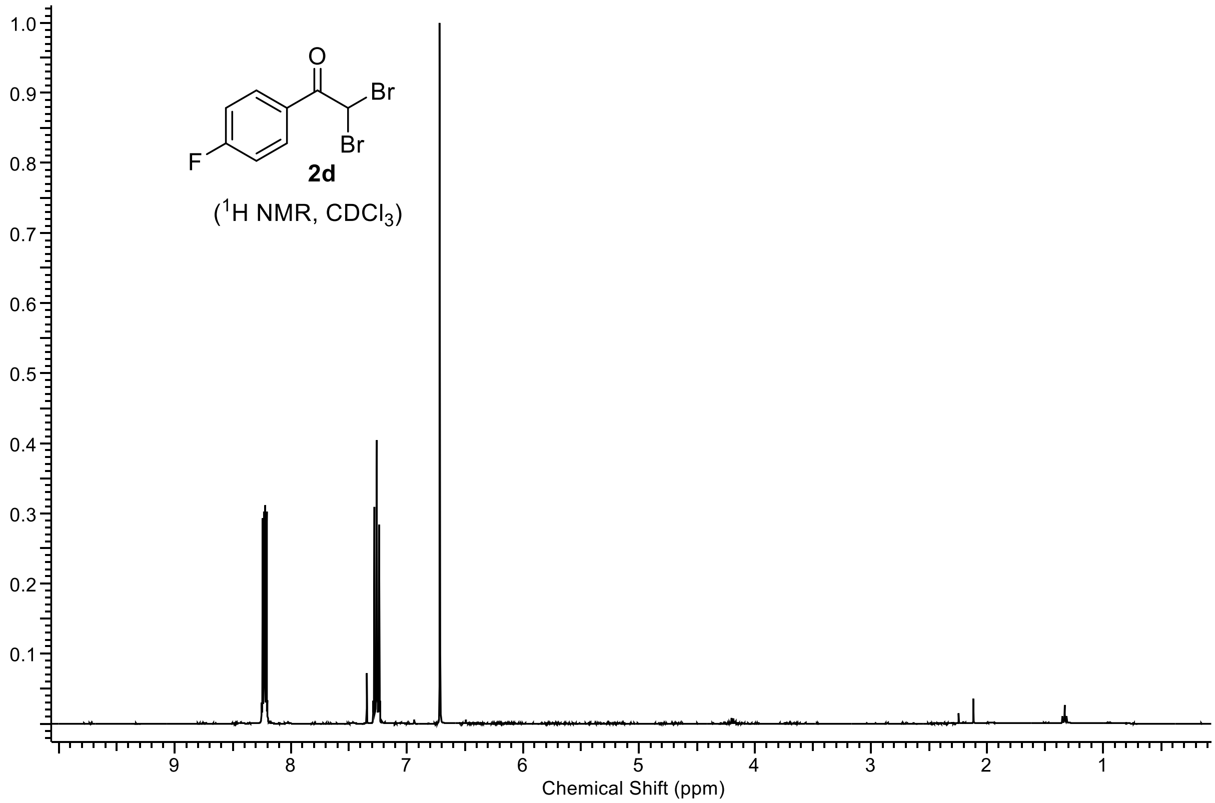
O
Br
Br
F
2d
(^{1}H NMR, CDCl$_3$)
1.0
0.9
0.8
0.7
0.6
0.5
0.4
0.3
0.2
0.1
9
8
7
6
5
4
3
2
1
Chemical Shift (ppm)

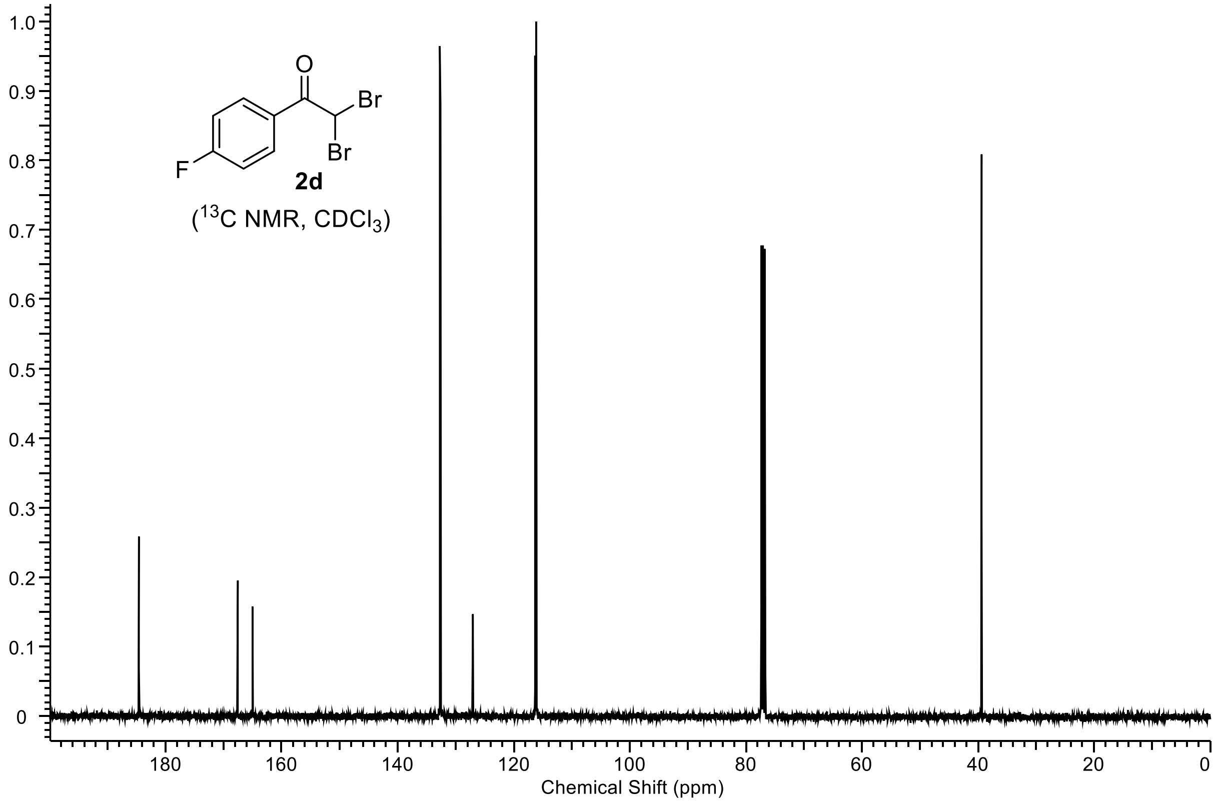
O
Br
Br
F
2d
(^{13}C NMR, CDCl$_3$)
1.0
0.9
0.8
0.7
0.6
0.5
0.4
0.3
0.2
0.1
0
180
160
140
120
100
80
60
40
20
0
Chemical Shift (ppm)

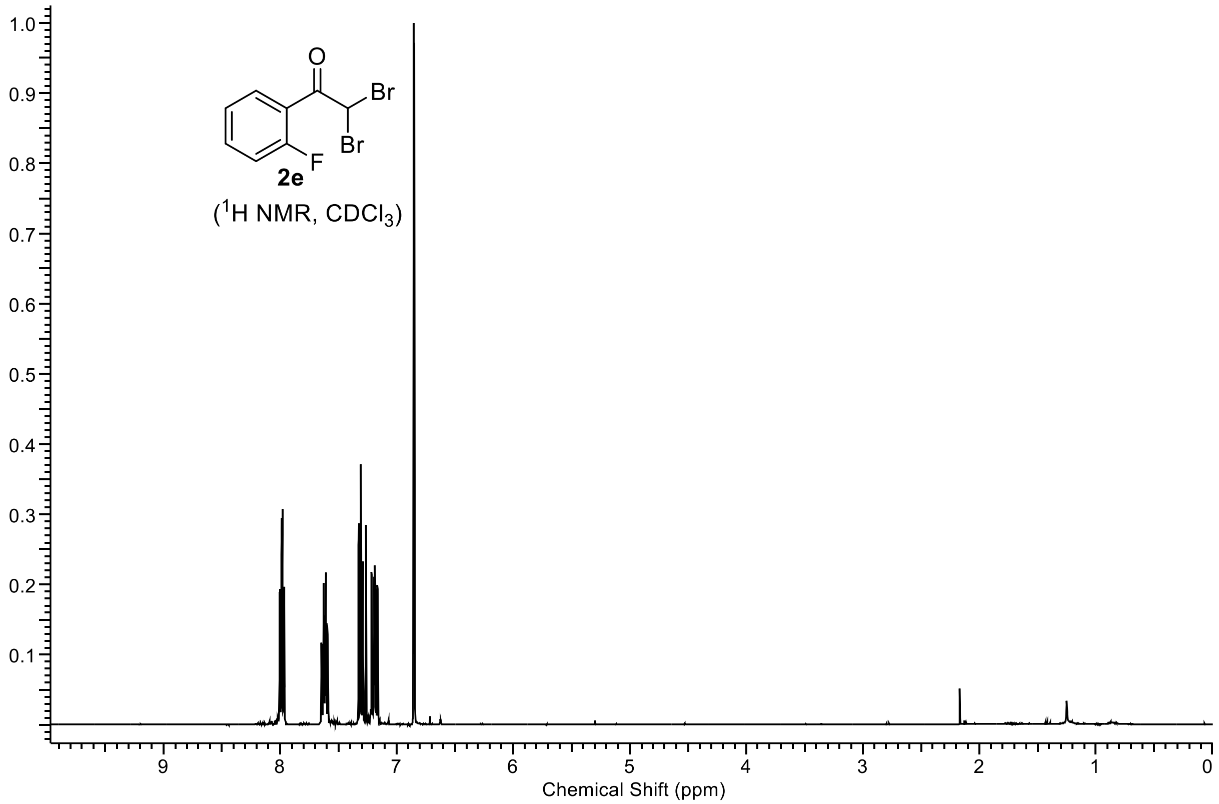

2e
(1H NMR, CDCl3)
Chemical Shift (ppm)

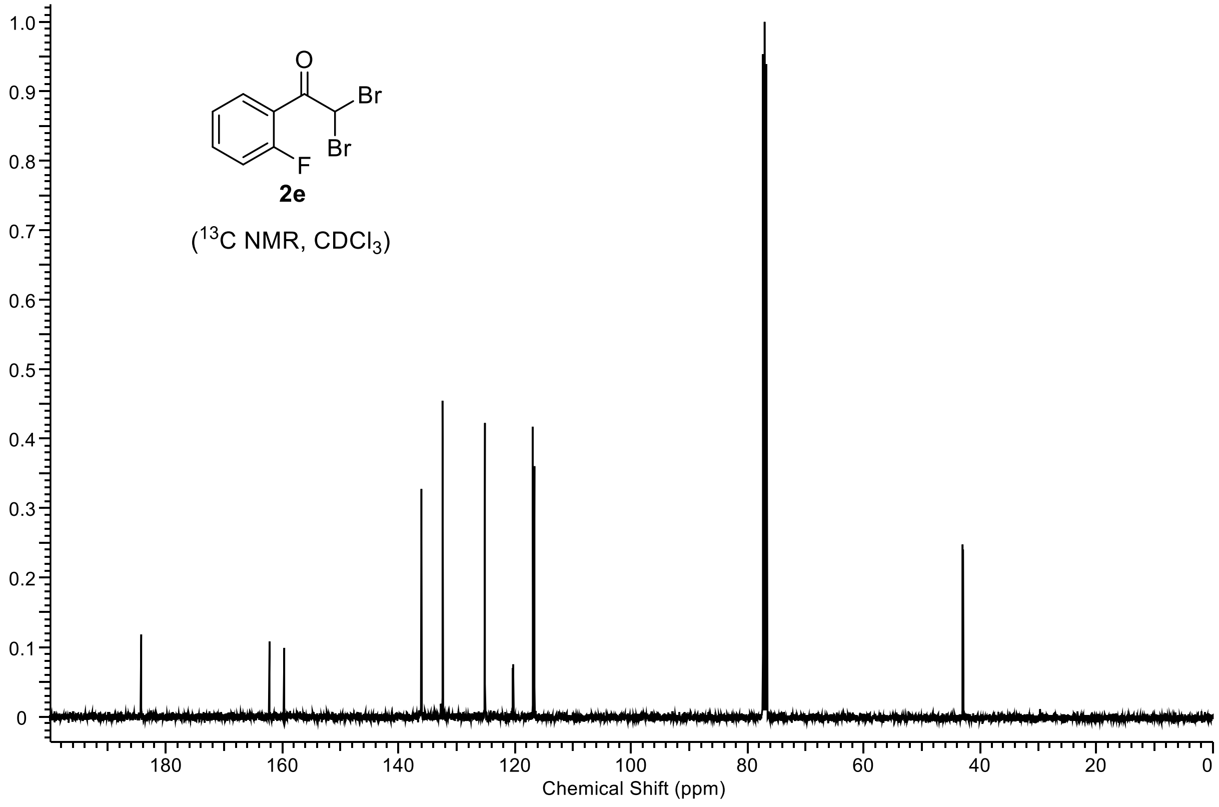

2e
(13C NMR, CDCl3)
Chemical Shift (ppm)

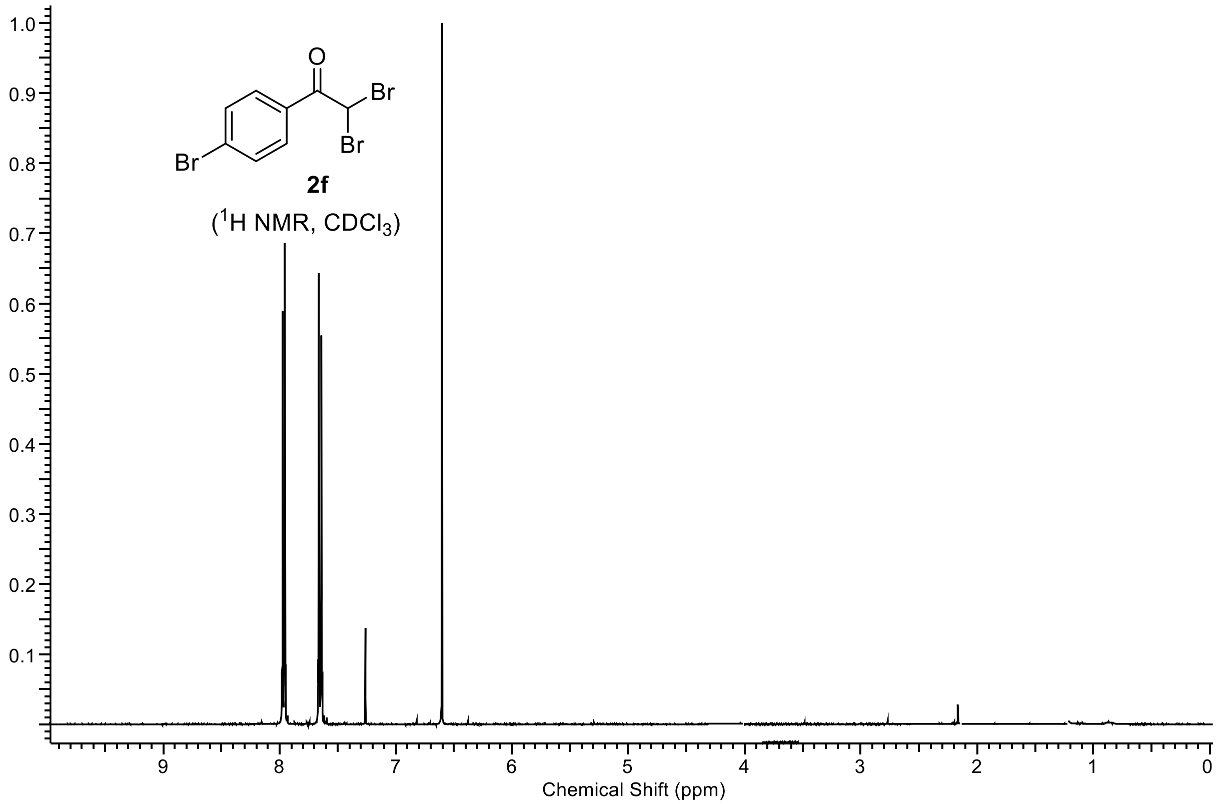

O
Br
Br
Br
2f
(^{1}H NMR, $CDCl_3$)
1.0
0.9
0.8
0.7
0.6
0.5
0.4
0.3
0.2
0.1
9
8
7
6
5
4
3
2
1
0
Chemical Shift (ppm)

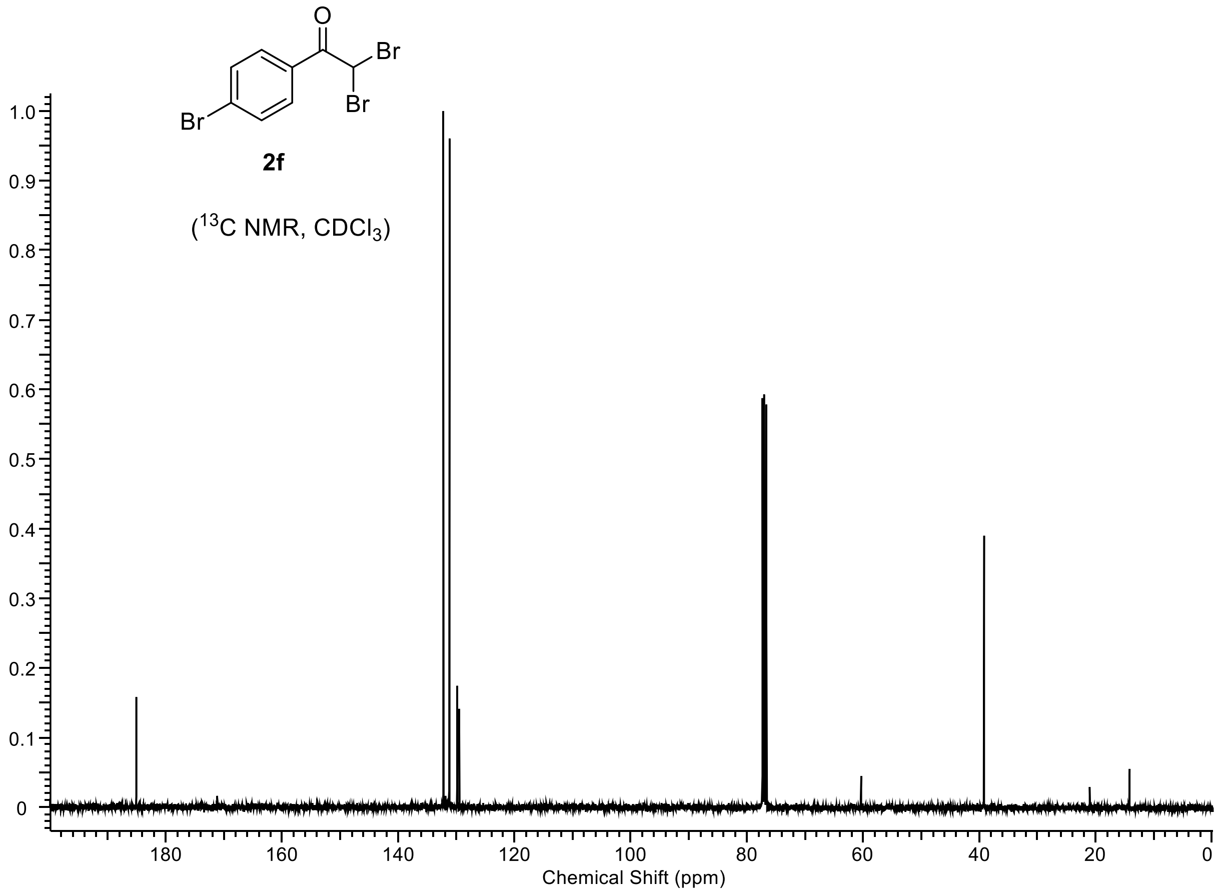

O
Br
Br
Br
2f
(^{13}C NMR, $CDCl_3$)
1.0
0.9
0.8
0.7
0.6
0.5
0.4
0.3
0.2
0.1
0
180
160
140
120
100
80
60
40
20
0
Chemical Shift (ppm)

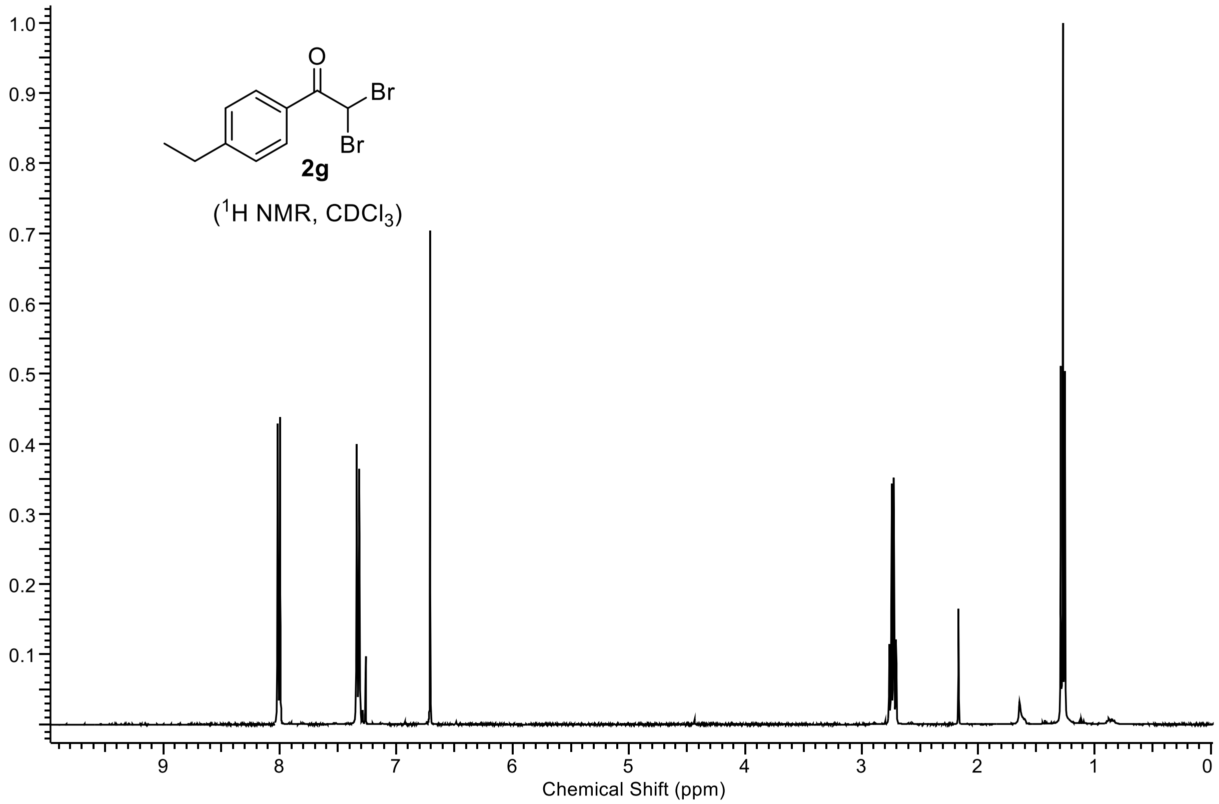

O
Br
Br
2g
(^{1}H NMR, CDCl$_3$)
Chemical Shift (ppm)

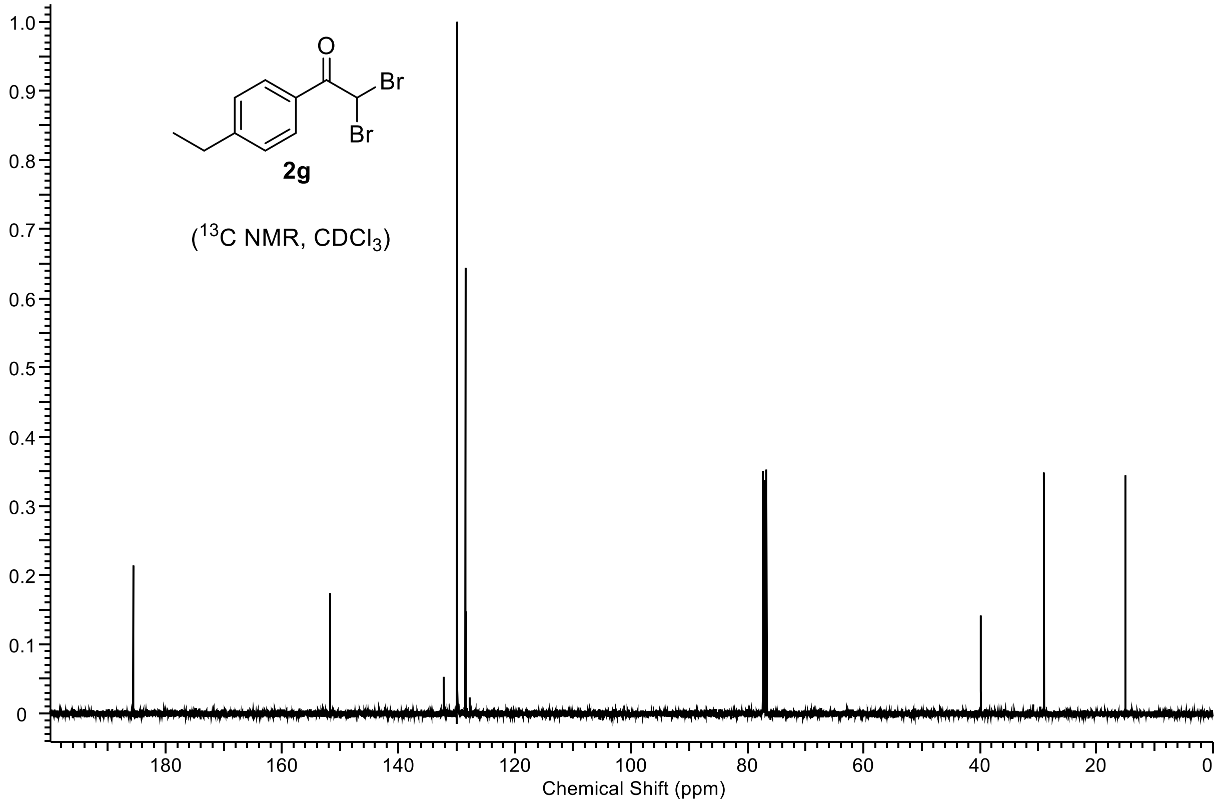

O
Br
Br
2g
(^{13}C NMR, CDCl$_3$)
Chemical Shift (ppm)

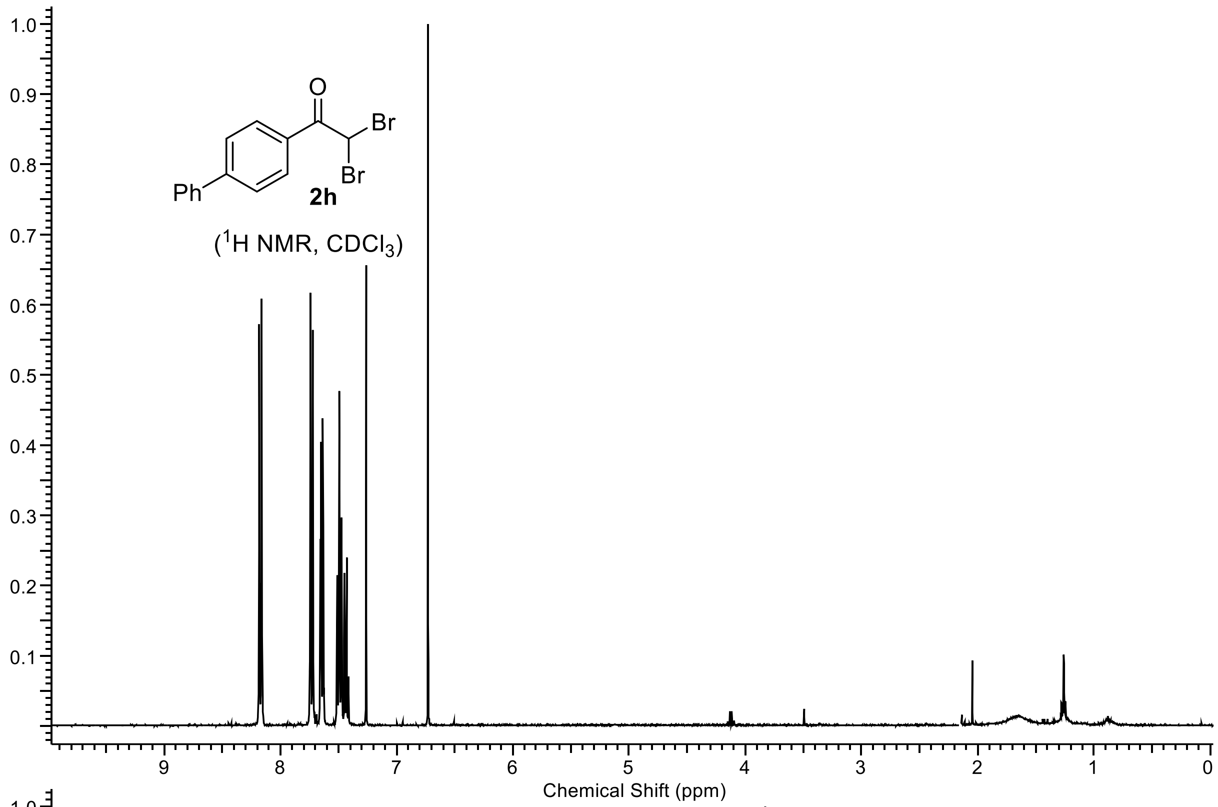

O
Br
Br
Ph
2h
(¹H NMR, CDCl3)
Chemical Shift (ppm)

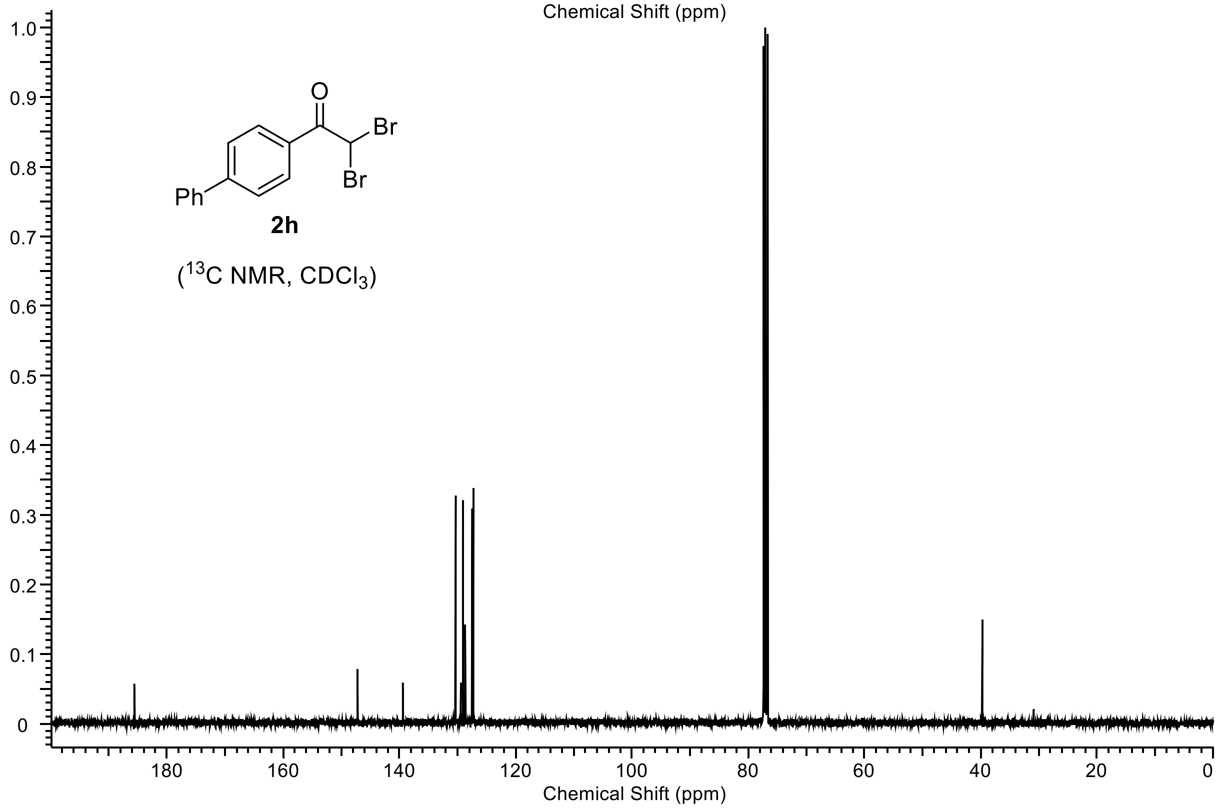

O
Br
Br
Ph
2h
(¹³C NMR, CDCl3)
Chemical Shift (ppm)

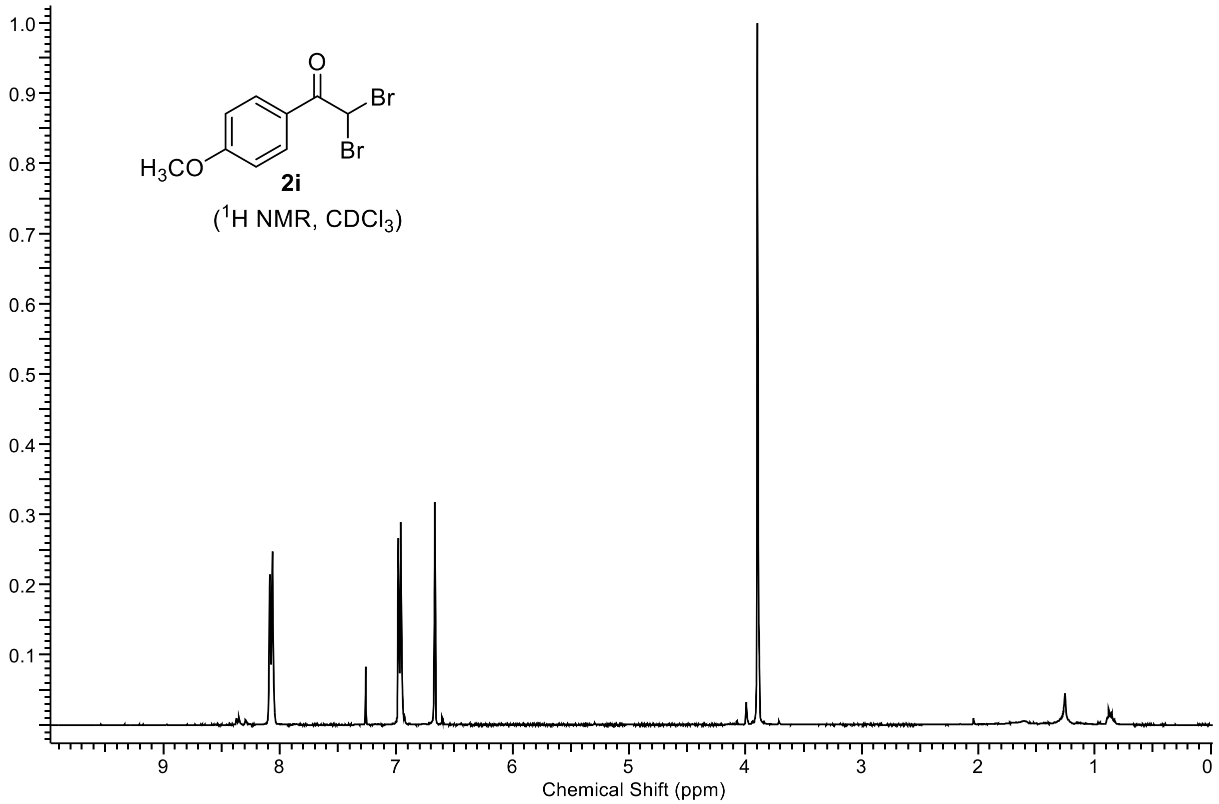
Br
Br
H3CO
O
2i
(1H NMR, CDCl3)
Chemical Shift (ppm)

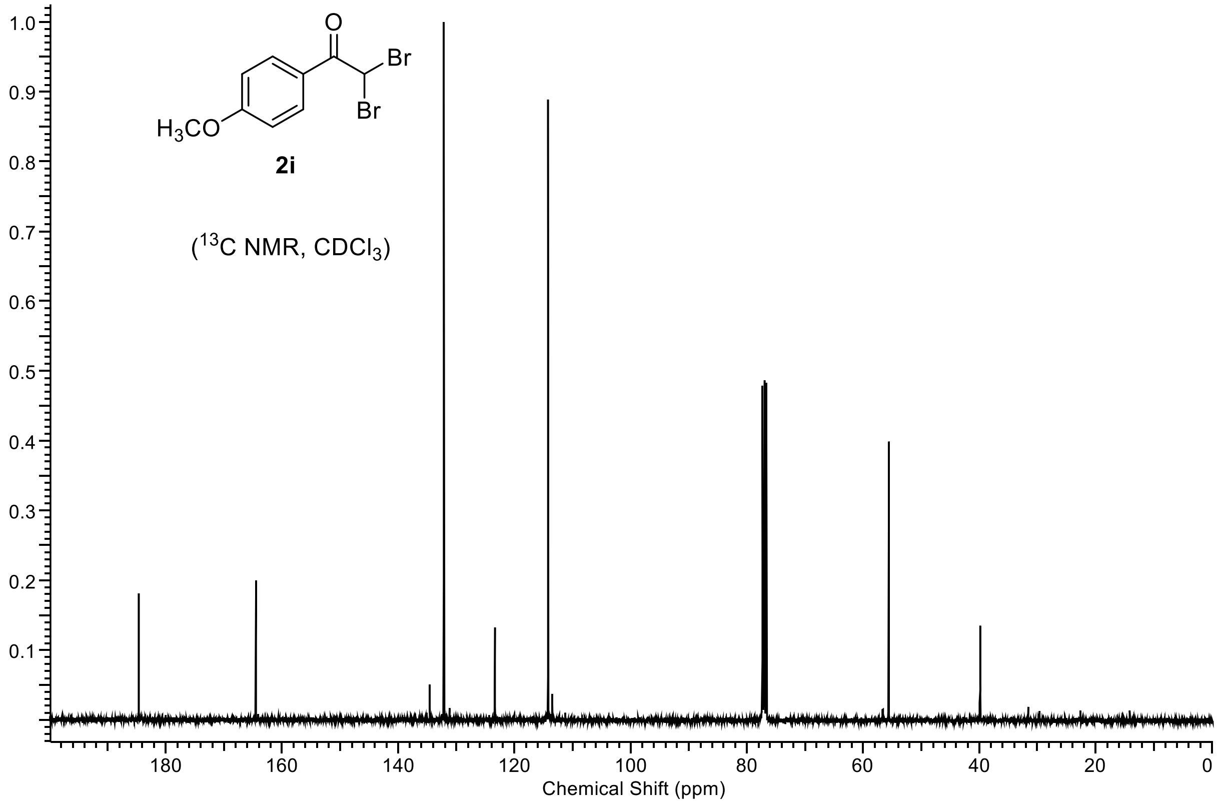
Br
Br
H3CO
O
2i
(13C NMR, CDCl3)
Chemical Shift (ppm)

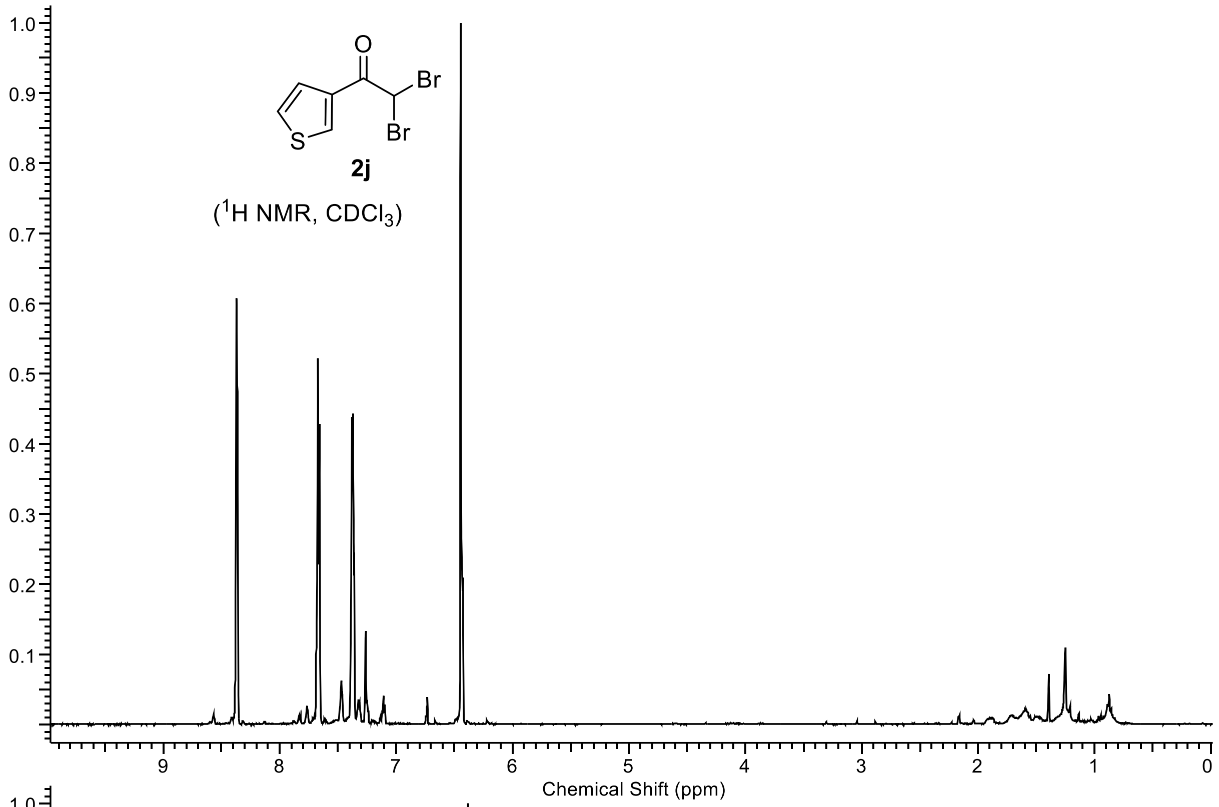

O
Br
S
Br
2j
(^{1}H NMR, $CDCl_3$)
Chemical Shift (ppm)

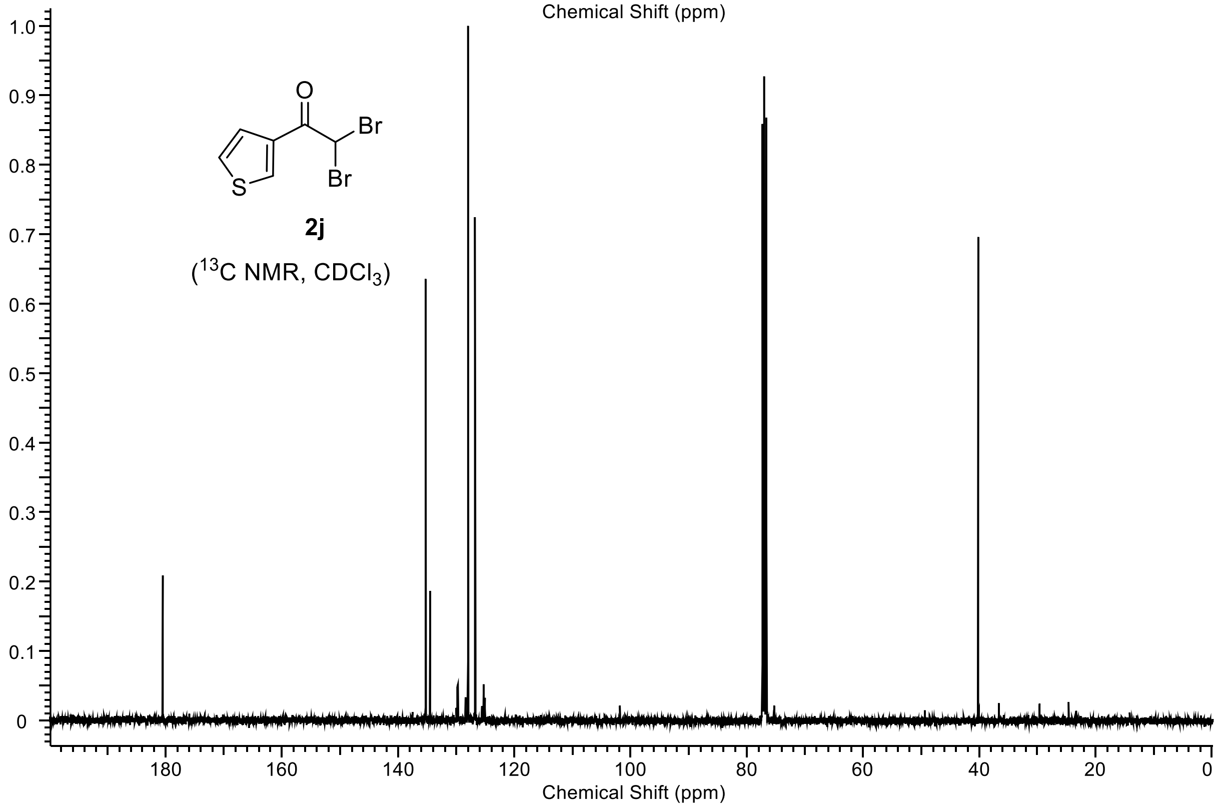

O
Br
S
Br
2j
(^{13}C NMR, $CDCl_3$)
Chemical Shift (ppm)

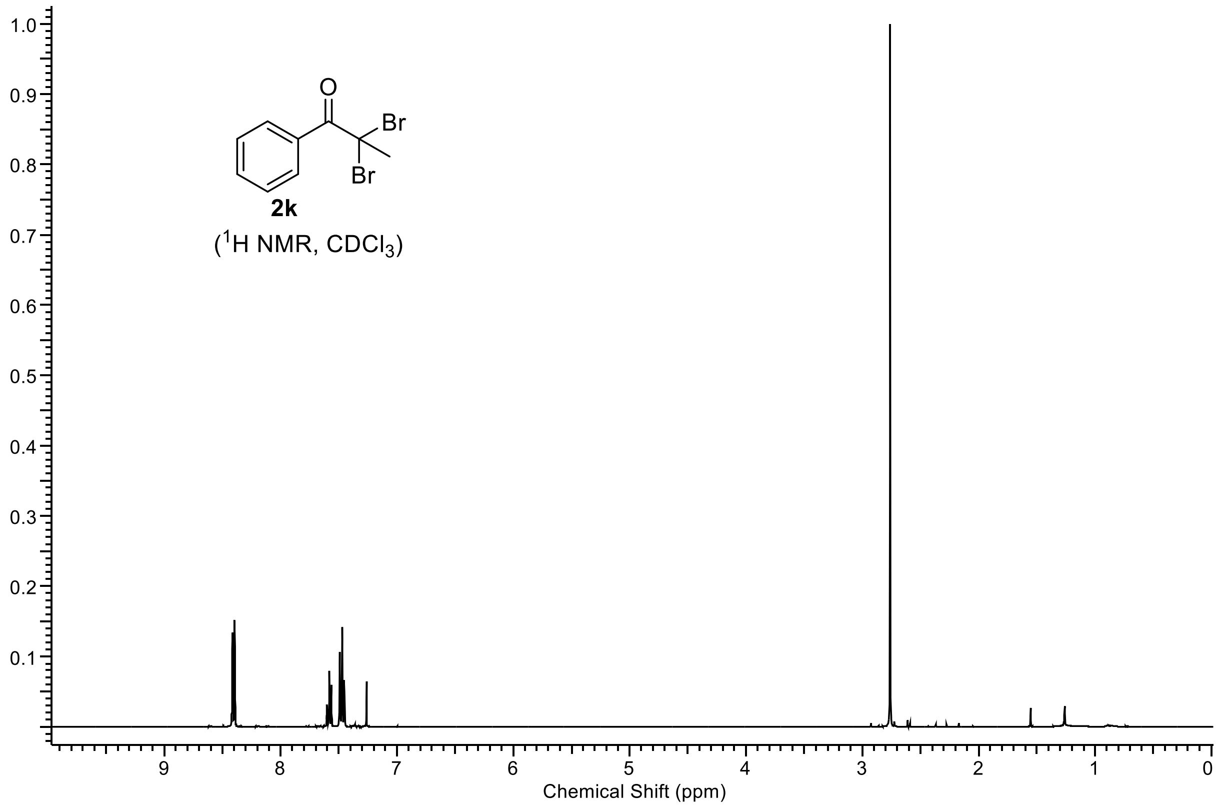

O
Br
Br
2k
(^{1}H NMR, CDCl$_3$)
Chemical Shift (ppm)

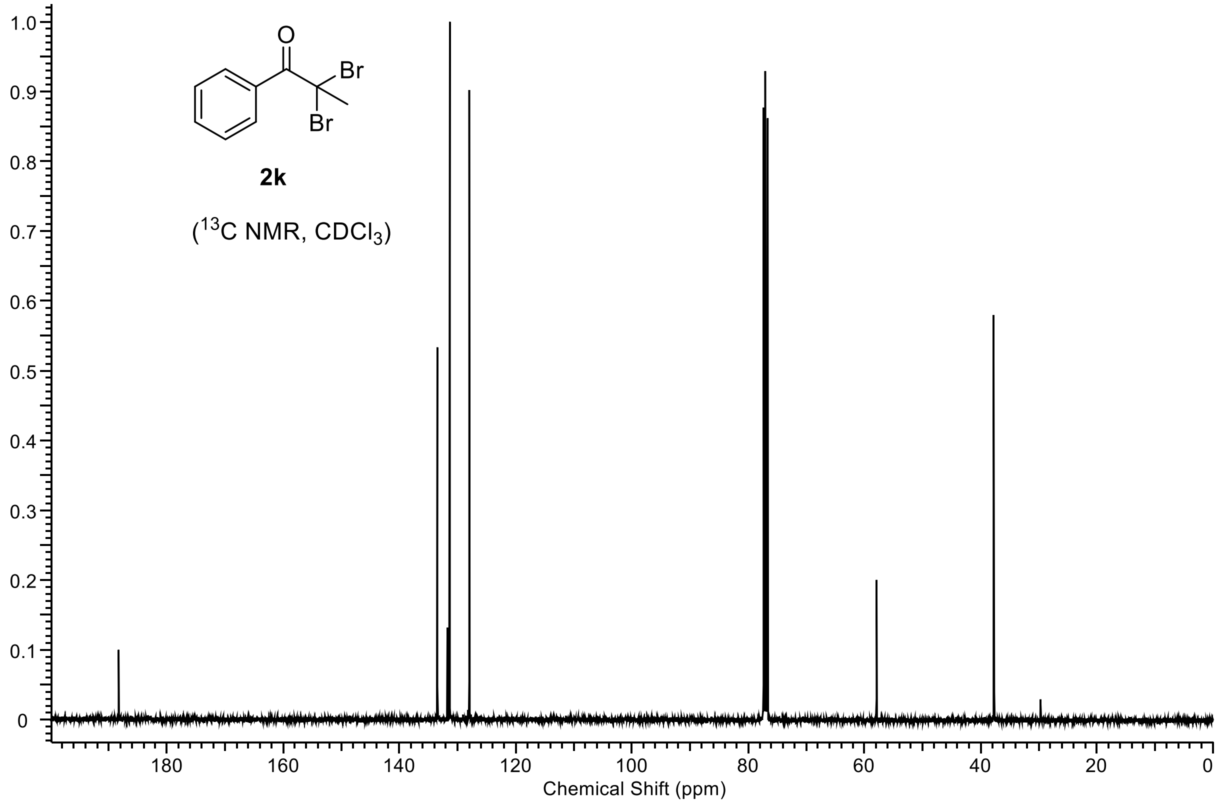

O
Br
Br
2k
(^{13}C NMR, CDCl$_3$)
Chemical Shift (ppm)

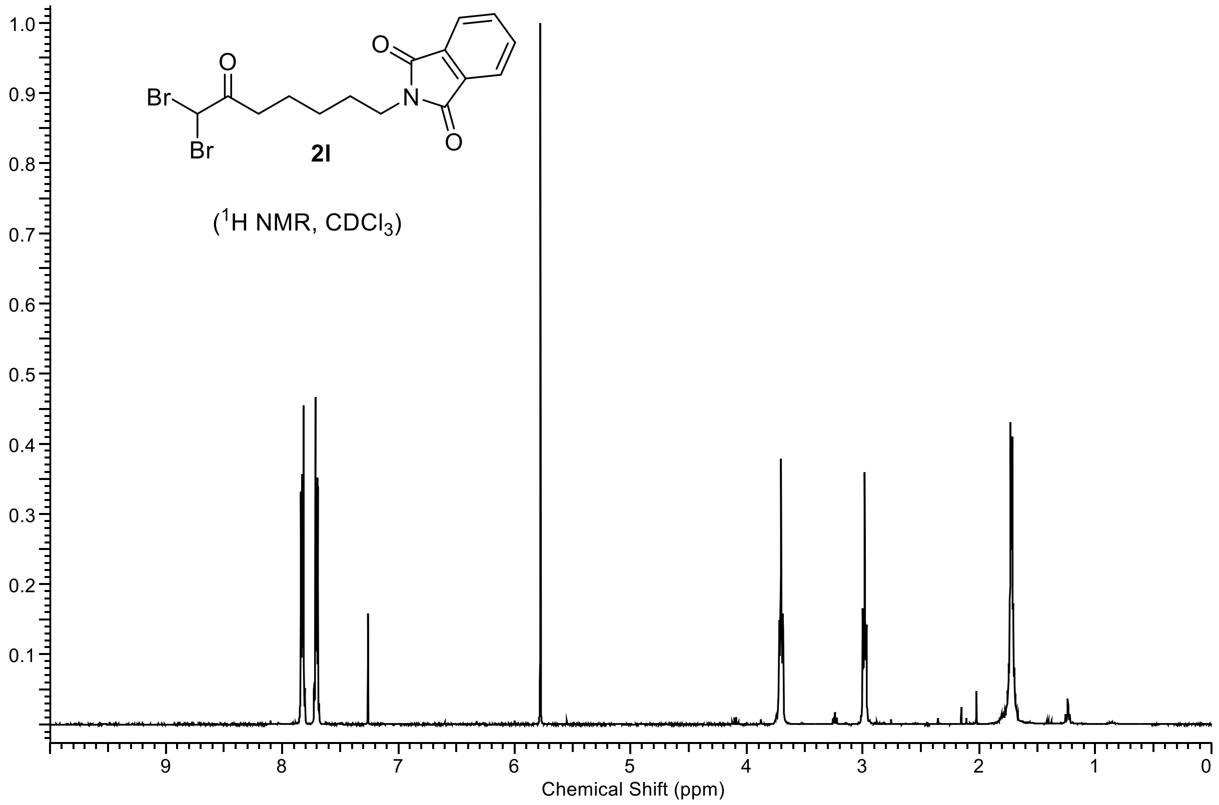

Br
O
Br
N
O
O
2l
(1H NMR, CDCl3)
Chemical Shift (ppm)

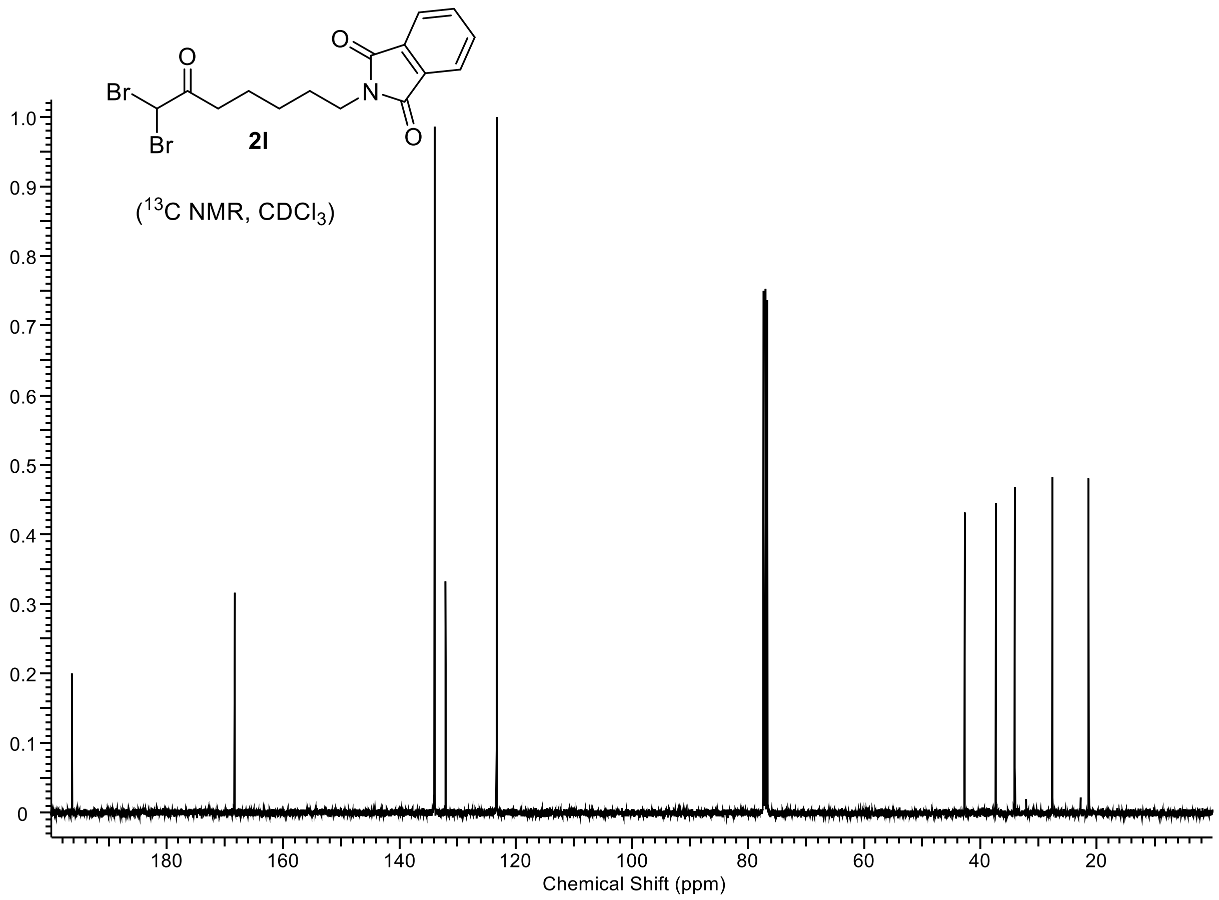

Br
O
Br
N
O
O
2l
(13C NMR, CDCl3)
Chemical Shift (ppm)

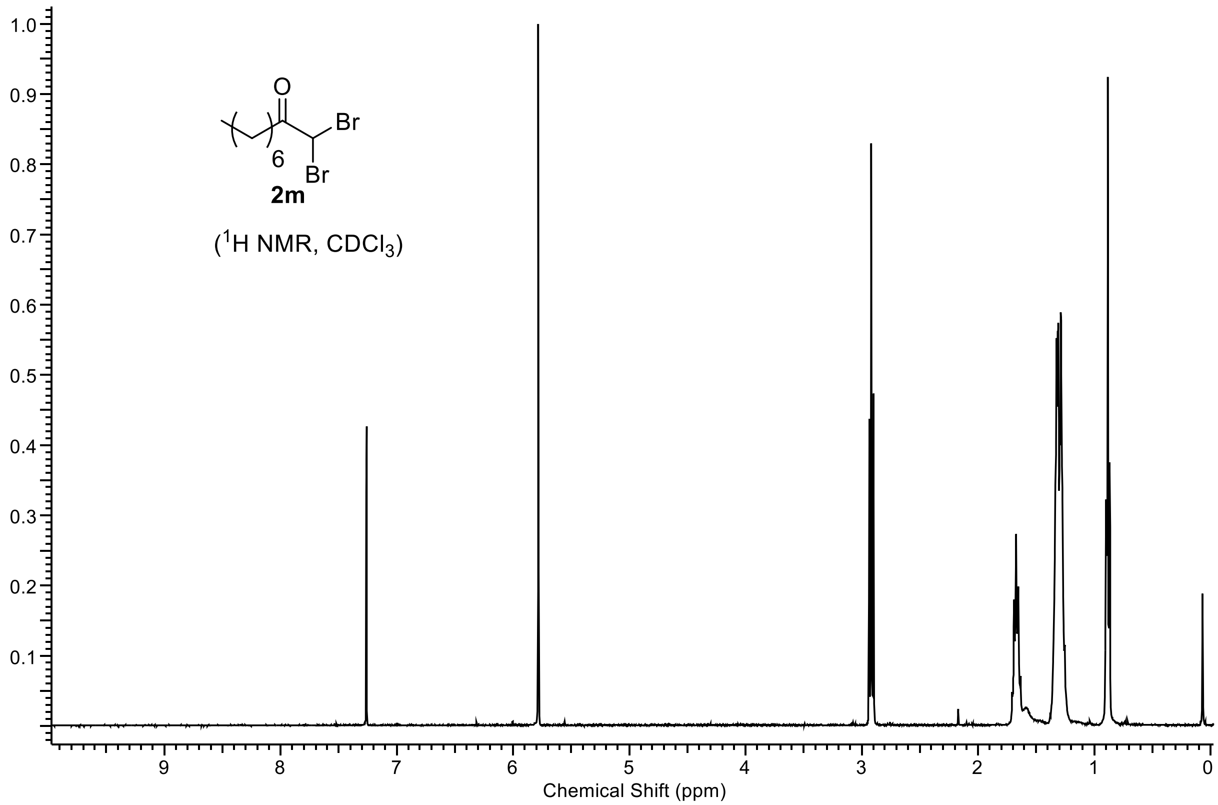
O
Br
6
Br
2m
(1H NMR, CDCl3)
Chemical Shift (ppm)

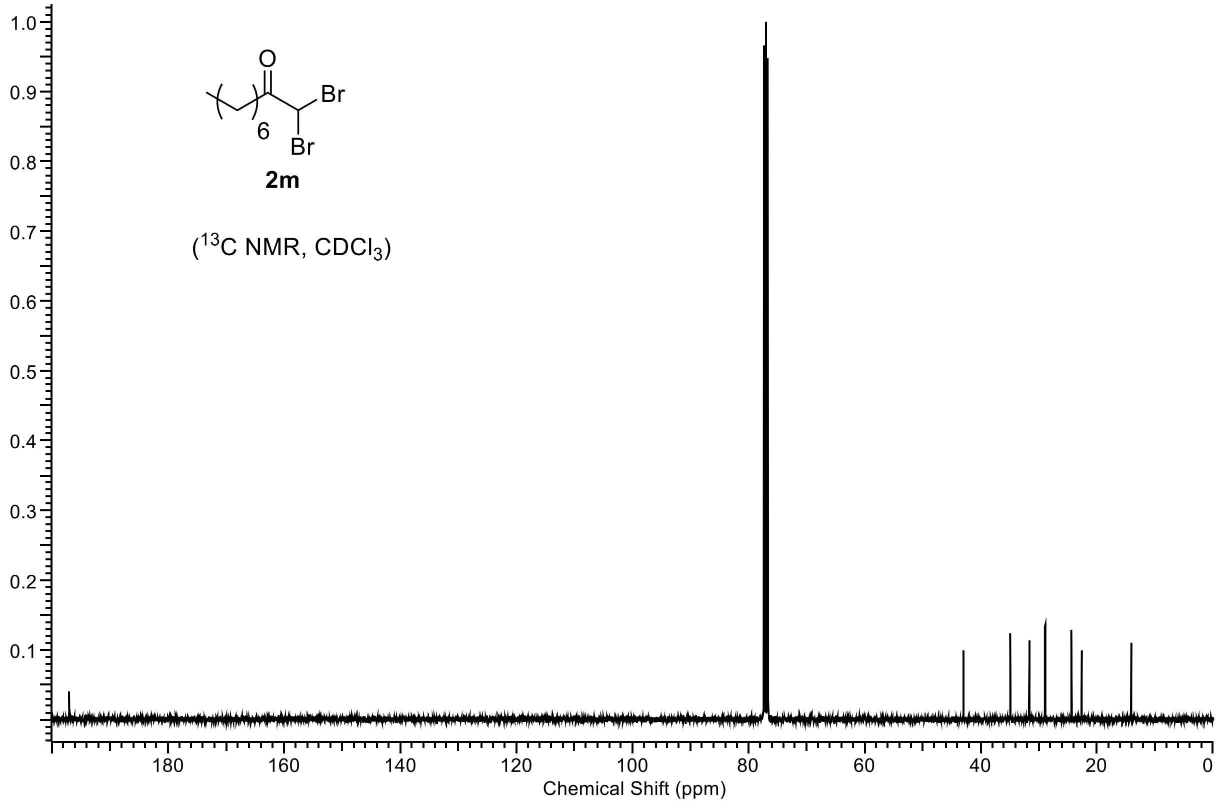
O
Br
6
Br
2m
(13C NMR, CDCl3)
Chemical Shift (ppm)

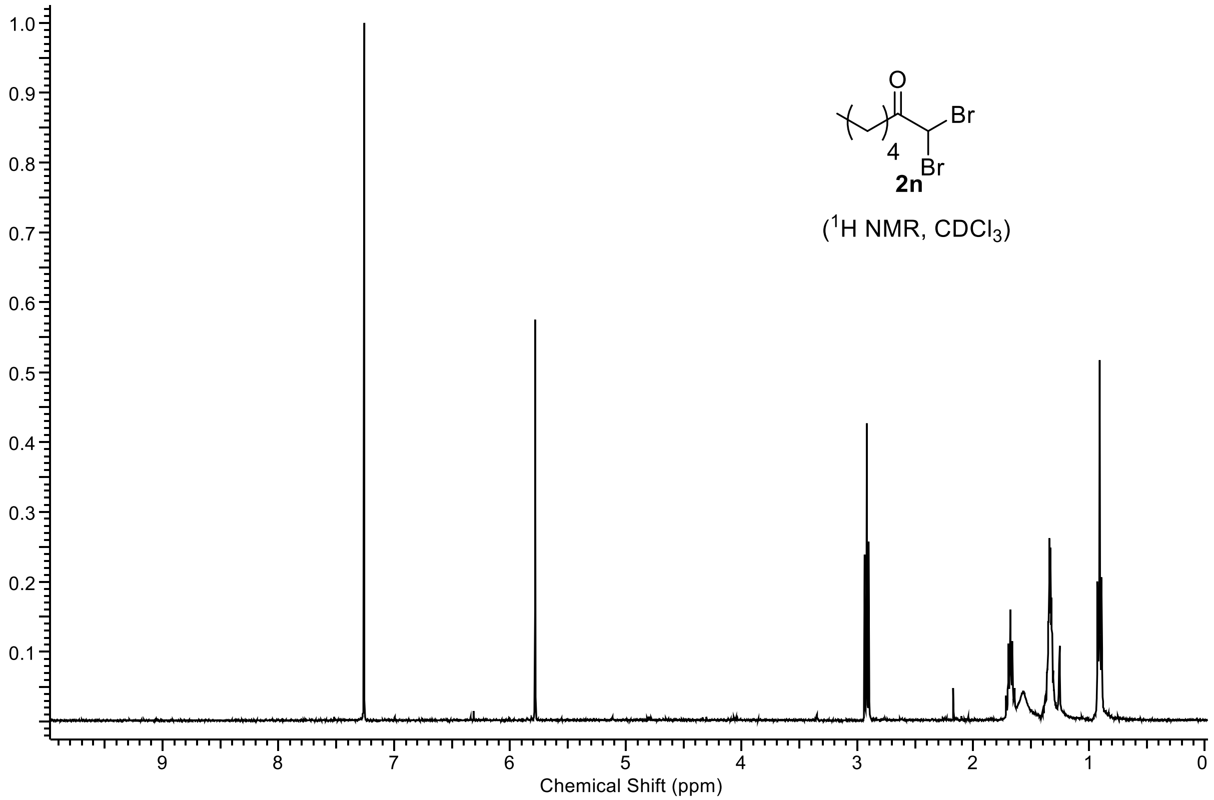

O
Br
4
Br
2n
(^{1}H NMR, CDCl$_3$)
1.0
0.9
0.8
0.7
0.6
0.5
0.4
0.3
0.2
0.1
9
8
7
6
5
4
3
2
1
0
Chemical Shift (ppm)

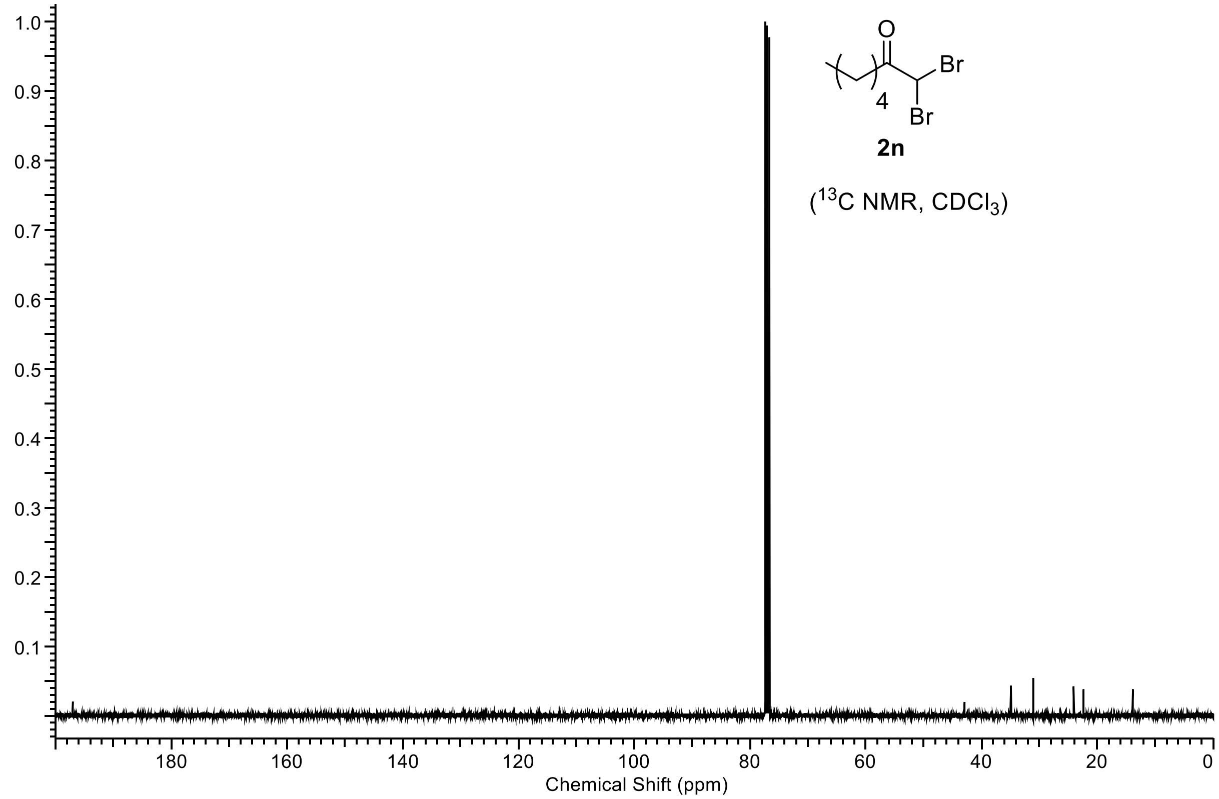

O
Br
4
Br
2n
(^{13}C NMR, CDCl$_3$)
1.0
0.9
0.8
0.7
0.6
0.5
0.4
0.3
0.2
0.1
180
160
140
120
100
80
60
40
20
0
Chemical Shift (ppm)

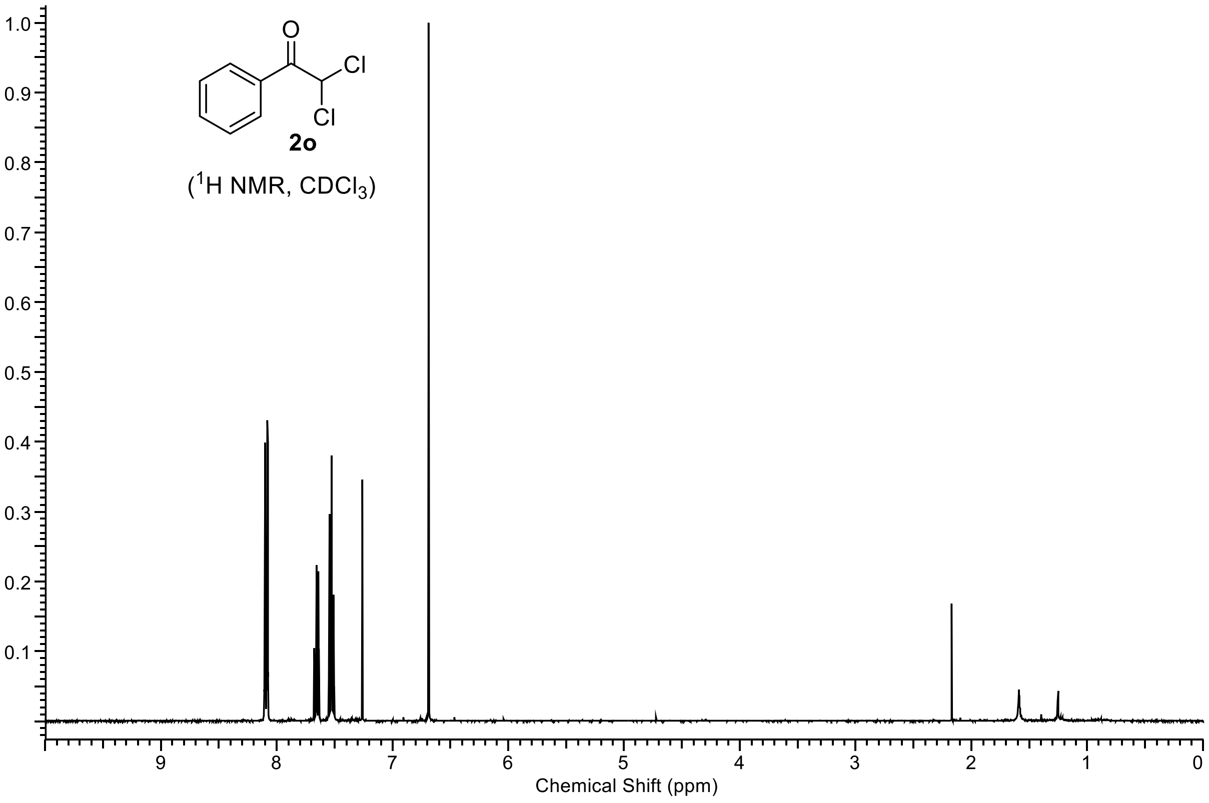

O
Cl
Cl
2o
(^{1}H NMR, CDCl$_3$)
1.0
0.9
0.8
0.7
0.6
0.5
0.4
0.3
0.2
0.1
9
8
7
6
5
4
3
2
1
0
Chemical Shift (ppm)

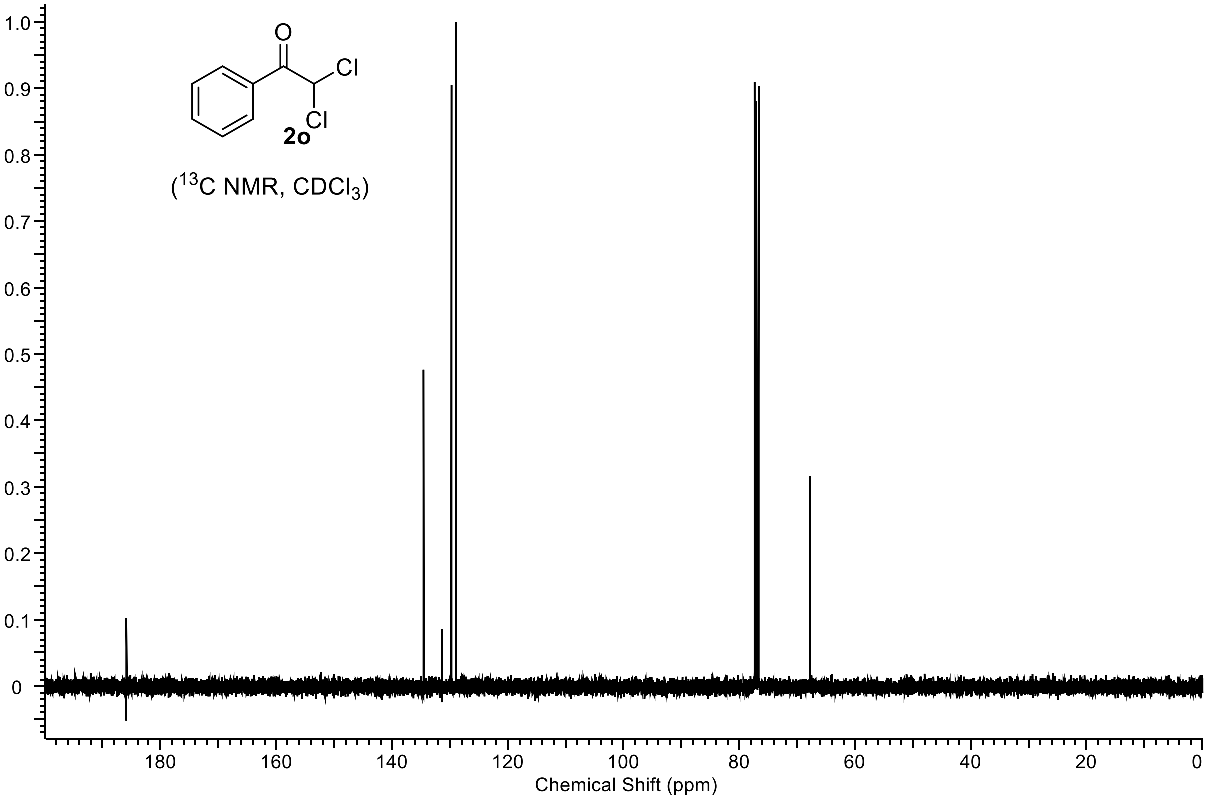

O
Cl
Cl
2o
(^{13}C NMR, CDCl$_3$)
1.0
0.9
0.8
0.7
0.6
0.5
0.4
0.3
0.2
0.1
0
180
160
140
120
100
80
60
40
20
0
Chemical Shift (ppm)

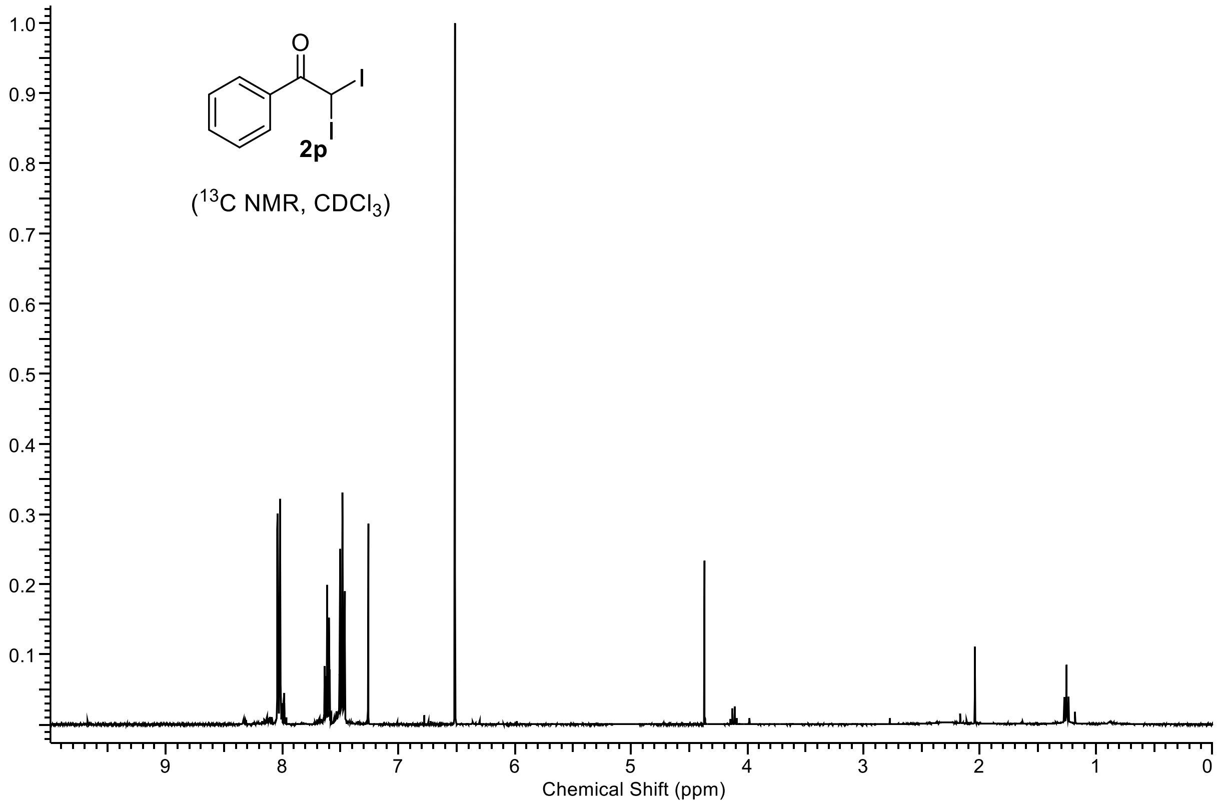

2p
(^{13}C NMR, $CDCl_3$)
Chemical Shift (ppm)

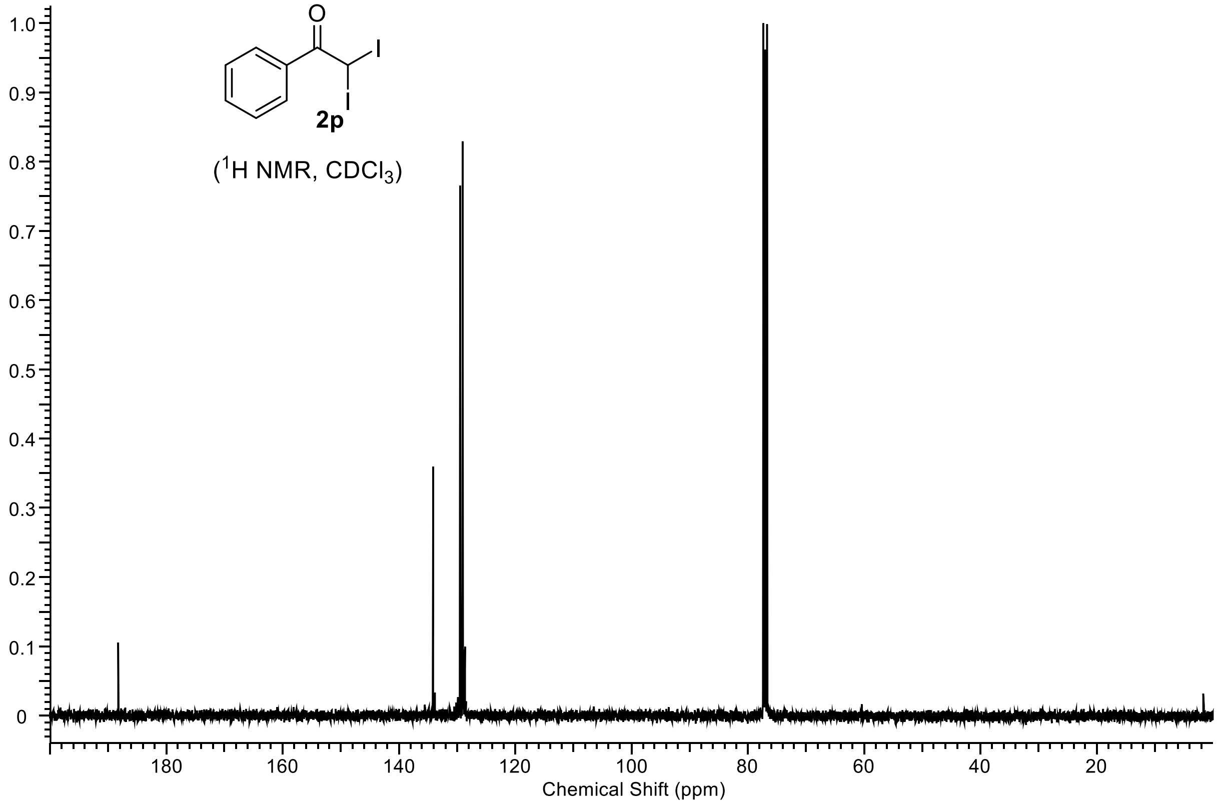

2p
(^{1}H NMR, $CDCl_3$)
Chemical Shift (ppm)
